# Cell compensatory responses of fungi to damage of the cell wall induced by Calcofluor White and Congo Red with emphasis on *Sporothrix schenckii* and *Sporothrix globosa*. A review

**DOI:** 10.3389/fcimb.2022.976924

**Published:** 2022-09-23

**Authors:** Jorge A. Ortiz-Ramírez, Mayra Cuéllar-Cruz, Everardo López-Romero

**Affiliations:** Departamento de Biología, División de Ciencias Naturales y Exactas, Universidad de Guanajuato, Guanajuato, Mexico

**Keywords:** *Sporothrix*, glucosamine-6-phosphate synthase, cell wall biogenesis, compensatory responses, cell signaling pathways

## Abstract

The cell wall (CW) of fungi exhibits a complex structure and a characteristic chemical composition consisting almost entirely of interacting crystalline and amorphous polysaccharides. These are synthesized by a number of sugar polymerases and depolymerases encoded by a high proportion of the fungal genome (for instance, 20% in *Saccharomyces cerevisiae*). These enzymes act in an exquisitely coordinated process to assemble the tridimensional and the functional structure of the wall. Apart from playing a critical role in morphogenesis, cell protection, viability and pathogenesis, the CW represents a potential target for antifungals as most of its constituents do not exist in humans. Chitin, β-glucans and cellulose are the most frequent crystalline polymers found in the fungal CW. The hexosamine biosynthesis pathway (HBP) is critical for CW elaboration. Also known as the Leloir pathway, this pathway ends with the formation of UDP-N-GlcNAc after four enzymatic steps that start with fructose-6-phosphate and L-glutamine in a short deviation of glycolysis. This activated aminosugar is used for the synthesis of a large variety of biomacromolecules in a vast number of organisms including bacteria, fungi, insects, crustaceans and mammalian cells. The first reaction of the HBP is catalyzed by GlcN-6-P synthase (L-glutamine:D-fructose-6-phosphate amidotransferase; EC 2.6.1.16), a critical enzyme that has been considered as a potential target for antifungals. The enzyme regulates the amount of cell UDP-N-GlcNAc and in eukaryotes is feedback inhibited by the activated aminosugar and other factors. The native and recombinant forms of GlcN-6-P synthase has been purified and characterized from both prokaryotic and eukaryotic organisms and demonstrated its critical role in CW remodeling and morphogenesis after exposure of some fungi to agents that stress the cell surface by interacting with wall polymers. This review deals with some of the cell compensatory responses of fungi to wall damage induced by Congo Red and Calcofluor White.

## Introduction

### The cell wall

The CW is a highly dynamic and plastic structure whose heterogenous composition is essential for cell viability, morphogenesis and pathogenesis. This outermost structure represents a barrier against environmental physical and chemical factors, modulates permeability and protects the cell from mechanical or osmotic stress. Its composition and structural organization are regulated by environmental factors through CW adhesins and a diversity of receptors that once activated trigger a number of signaling pathways aimed to maintain the cellular homeostasis through compensatory responses (Gow et al., 2017; Beauvais and Latgé, 2018; Ibe and Munro, 2021). The fungal CW has attracted the attention as a potential target of antifungals as their components do not exist in the humans; yet there has been a little success in this context since only echinocandin, a blocker of β-1,3-glucan synthase, has been used in human infections (Cortes et al., 2019; Ibe and Munro, 2021).

#### Composition, structure, and biogenesis of the fungal CW

Polysaccharides of the fungal wall constitute a sort of central, resistant core consisting of diverse proteins and gel carbohydrates whose interactions form a strong, flexible and chemically diverse structure (Arroyo et al., 2016; Gow et al., 2017; Carvalho et al., 2021) that results in a lack of homology among fungi, plant and bacteria (Coronado et al., 2007). Accordingly, the CW can be defined as a complex and specific highly dynamic layer whose biosynthesis, remodelation and functional maintenance involve a great number of diverse enzymes and signaling pathways.

Polysaccharides such as chitin and β-glucan, two common components of the fungal CW, are coordinately synthesized by transmembrane enzymatic complexes consisting of chitin and glucan synthases (Schuster et al., 2016) that are transported inactive in vesicles and activated once they get in contact with the membrane. This is different to the synthesis of glycoconjugates whose assembly occurs in the rough endoplasmic reticulum and in the Golgi membrane complex where they interact with proteins through a canonical secretory pathway (Kollaír et al., 1997; Schuster et al., 2016). Since chitin and β-glucan are synthesized from the corresponding activated sugars UDP-N-GlcNAc and UDP-Glc, respectively, this implies that enzymes in the metabolic pathways leading to the formation of these activated precursors also play a determinant role in wall biogenesis and modulation (Free, 2013; Gow et al., 2017).

In most fungi, the CW exhibits two layers. The innermost one shows a more conserved structure than the outermost layer which is more complex and exhibits a great diversity among different organisms (Gow et al., 2017). The inner wall is made up mainly of branched β-1,3- and β-1,6-glucans as well as α-1,3- and α-1,6-glucan that represent 50-60% of the wall dry weight. On the other hand, chitin, the homopolymer made of linear chains of acetylglucosamine units and the most ancient CW polysaccharide, constitutes 1-2% in yeast but can account for 10-20% in filamentous fungi. Together, interchained glucan and chitin form a very dynamic, tridimensional wire mesh whose structural assembly resists the hydrostatic pressure imposed by the plasma membrane and the cytoplasm (Kollár et al., 1997; Klis et al., 2002; Latgé, 2010; Gow et al., 2017). The outermost layer, which is adaptable to the physiology of the organism, is more diverse and variable among organisms and it consists mainly of proteins that represent 30-50% and 20-30% of the wall dry weight in yeast and hyphae, respectively, and are commonly found O- and N-linked to oligosaccharides forming glycoproteins.

Other wall components are proteins attached to membranes through glycosylphosphatidylinositol forming what it is known as GPI anchors. To date, a number of proteins anchored to the membrane by GPI have been identified in various eukaryotes from protozoa and fungi to human beings where they exhibit diverse structures depending on the bound protein and the organism. They regulate the mobility of anchored proteins due to their interaction with the membrane bilayer (Fein et al., 1993) and carry out several functions such as the defense of the complement system, membrane receptors and cell protection (Lucero and Robbins, 2004; Nohe et al., 2006). The presence of proteins with internal repeats (Pir proteins) has been described in the CW of some organisms such as *S. cerevisiae* (Klis et al., 2002). These are used as anchor proteins in the cell surface that significantly increase the proteins to be displayed (Yang et al., 2014). Other functions attributed to Pir proteins are the masking and immune blocking absorption of molecules, cell adhesion and signal transduction (Carvalho et al., 2021; Ibe and Munro, 2021).

### *Sporothrix* species as models to study CW structure and function

Species of the pathogenic clade of the *Sporothrix* genus have been used to investigate a number of functions related with the physiology and metabolism of the fungus. These include aspects such as differentiation/dimorphism, pathogenesis, the enzymology and molecular mechanisms of wall glycoprotein assembly, the search of surface antigens, particularly adhesins, CW biogenesis and regulation, and most recently the cell compensatory responses to damage of the cell surface. *Sporothrix* species include *S. schenckii* sensu stricto, *S. brasiliensis*, *S. globosa*, and *S. luriei* that cause a mycosis known as sporotrichosis, a disease of low morbidity and mortality of humans and some animals such as dogs and cats (Romeo and Criseo, 2013; Zhang et al., 2015; Huang et al., 2016; Lopes-Bezerra et al., 2018a). These species exhibit different levels of virulence, variable forms of transmission and geographic distribution (Lopes-Bezerra et al., 2018a) and are dimorphic organisms whose morphology depends on temperature, pH and other factors. Thus, at 25-28°C and an acidic pH, they develop as hyphae which is considered the saprophytic phase whereas at 32-37°C they grow as yeast or cigar-shaped cells, which is the morphotype frequently isolated from infected tissues (Rodríguez-del-Valle et al., 1983; López-Romero et al., 2011; Lopes-Bezerra et al., 2018a). Human sporotrichosis is caused by the traumatic acquisition of the fungus and it progresses as skin nodules and lymphocutaneous lesions (Travassos, 1985), usually following a mild course though it can disseminate in immunodeficient patients (Heller and Fuhrer, 1991; Durden and Elewski, 1997; Al-Tawfiq and Wools, 1998). In fact, recent years have witnessed an increasing number of infections caused by dimorphic fungi, an issue of great concern in public health. Among these, sporotrichosis caused by *S. schenckii* has nowadays the highest morbidity in immunocompromised patients, particularly in areas where the fungus is endemic and can lead to outbreaks of this mycosis (López-Romero et al., 2011; Barros et al., 2011; Chakrabarti et al., 2015; Conceição-Silva and Morgado, 2018; Queiroz-Telles et al., 2019). The dimorphism-pathogenesis relationship of *S. schenckii* was recently demonstrated in a patient with pulmonary disseminated sporotrichosis by comparing gene expression in the mycelium and yeast phases by RNA deep sequencing. Results of the transcriptomic analysis indicated that morphological transition of the virulent isolate depends on temperature and is closely associated with adaptation to stress, growth and development, signal control, adhesion and dissemination (He et al., 2021).

#### CW composition of *Sporothrix*

The CW of *S. schenckii* is made up of glucans bearing β1-3, β1-6 and β1-4 bonds, chitin and a peptidorhamnomannan (Lloyd and Bitoon, 1971; Previato et al., 1979; Lopes-Bezerra, 2011; Lopes-Bezerra et al., 2018b). A comparison of the chemical composition and structure of the CW of *S. schenckii* and *S. brasiliensis* revealed that both fungi exhibit a bilayered wall with the outermost layer containing a fibrillar peptidorhamnomannan component, while chitin, β-1,3- β-1,6-glucans and glycogen α-particles are present in the innermost layer in the proximity of the plasma membrane. Moreover, *S. brasiliensis* contains more chitin and rhamnomannan polymers (Lopes-Bezerra et al., 2018b). The presence of the latter components and the absence of α-glucans, commonly found in other dimorphic fungi, led Lopes-Bezerra´s group to propose a new model for the wall of these species. More recently, the CW composition of three strains of *S. schenckii* and two strains of *S. brasiliensis* exhibiting different levels of virulence in a murine model of infection were compared, confirming previous results for the species (Villalobos-Duno et al., 2021). These authors observed, however, an increase in the rhamnose/β-glucan ratio in the strains that correlated with an increase in virulence as well as structural differences in the rhamnomannan, thereby emphasizing the role of this polysaccharide in pathogenicity.

### Hexosamine biosynthetic pathway in eukaryotes and prokaryotes and the physiological relevance of GlcN-6-phosphate synthase

**Figure 1:**
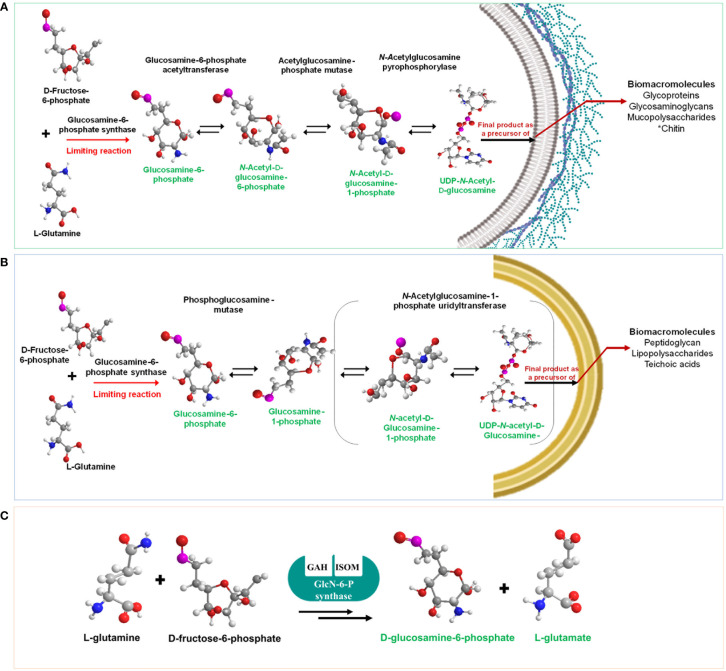
HBP in eukaryotes **(A)** and prokaryotes **(B)**. Notice that in prokaryotes, the pathway consists of only three enzyme reactions and that in both organisms GlcN-6-P synthase limits the production of UDP-N-acetylglucosamine, a precursor of a plethora of biomacromolecules in a great diversity of organisms. The reaction catalyzed by GlcN-6-P synthase is shown in **(C)** where the active centers of the enzyme are indicated. GAH, glutamine amidehydrolysing; ISOM, hexose phosphate-isomerizing.

The HBP, also known as the Leloir pathway (LP), describes the general metabolism of sugar units in polysaccharides using nucleotide-activated sugar precursors. This anabolic route is in fact a deviation of the glycolysis and was described in 1950 by the Nobel Prize winner Luis F. Leloir (Michal and Schomburg, 2013). The enzymes and mechanistics of the HBP have been described in detail elsewhere (Milewski, 2002; Milewski et al., 2006; Durand et al., 2008). The first committed step of this route is irreversibly catalyzed by GlcN-6-P synthase, which transforms fructose-6-phosphate into glucosamine-6-phosphate, using L-glutamine as the amino donor for which the enzyme exhibits an absolute specificity. Contrary to other amidotransferases, the enzyme cannot use ammonia as the amino donor. Depending on its origin, GlcN-6-P synthase has been named as GlmS, Gfa or Gfat. The substrates orderly bind to the enzyme and binding of fructose-6-phosphate precedes that of L-glutamine (Chmara et al., 1986; Badet et al., 1988). To date, it is the only enzyme that gives rise to GlcN-6-P. The molecular mechanism of the first reaction of HBP is complex and implies amide bond cleavage followed by ammonia transfer and isomerization of GlcN-6-phosphate into GlcN-1-phosphate which is finally converted into UDP-N-GlcNAc by three more reactions in eukaryotes (Bearne, 1996; Mi.lewski, 2002; Durand et al, 2008) (**Figure 1A**). Only three out of the four enzymes present in eukaryotes exist in prokaryotes, namely, GlcN-6-P-synthase, phosphoglucose mutase and the bifunctional enzyme N-acetylglucosamine-1-phosphate urdiltransferase that transfers the acetyl and uridyl residues (**Figure 1B**) (Smith et al., 1996; Smith, 2006; Barreteau et al., 2008; Assrir et al., 2014). The bacterial GlcN-6-P-synthase is a homodimer of 130-150 kDa whereas its eukaryotic counterpart is a homotetramer and exhibits a molecular mass of 320-340 kDa (Raczynska et al., 2007). GlcN-6-P-synthases from higher organisms share a sequence identity of 30-35% with their bacterial counterparts and all conserve the substrate domains suggesting a similar catalytic mechanism (**Figure 1C**) (Durand et al., 2008). Back in 1976, it was demonstrated that GlcN-6-P-synthase was specifically inhibited by anticapsin, a glutamine analog (Kenig et al., 1976), thereby opening the possibility of using the enzyme as a potential target for antimicrobial drugs (Milewski et al., 1988; Wojciechowski et al., 2005).

GlcN-6-P synthase is present in a great diversity of organisms (Milewski, 2002). The enzyme is extremely unstable; in fact, Prof. Milewski has defined it as a “nasty enzyme”. This property and in some cases the low amount present in the cell cytoplasm (for instance, in *C. albicans*, the enzyme represents less than 0.025% of cytosolic protein) have seriously limited its purification and characterization. In this context, the enzyme from *Escherichia coli* was purified to homogeneity and its structural and catalytic properties were documented (Badet et al., 1987; Teplyakov et al., 2001; Teplyakov et al., 2002; Durand et al., 2008).

Overexpression of the corresponding genes in different hosts has allowed the purification and characterization of GlcN-6-P-synthase from *S. cerevisiae* (Milewski et al., 1996) and *C. albicans* (Milewski et al., 1999; Sachadyn et al., 2000). The best studied enzyme in terms of the structural, functional and regulatory properties is by large that from *C. albicans* (Milewski et al., 1999). Moreover, the enzyme has been partially purified and characterized from other fungi such as *Neurospora crassa* (Ghosh et al., 1960), *Blastocladiella emersonii* (Norrman et al., 1975) and *Aspergillus nidulans* (Borgia, 1992). Other sources of GlcN-6-P synthase include human liver (Kikuchi and Tsuiki, 1976), pig (Zwierz et al., 1978) and mouse (Hosoi et al., 1978) tissues and plants (Vessal and Hassid, 1972). However, little is known about the enzyme in true dimorphic fungi such as *Sporothrix* species.

#### HBP and cellular morphogenesis

It has been demonstrated that operation of fungal HBP is important during morphological transitions such as the formation of the germ tube in *C. albicans*, a process that is paralleled by a 3-5-fold increase in the chitin content of the CW (Chattaway et al., 1968), a 4-fold increase in the specific activity of GlcN-6-P synthase (Chiew et al., 1980) and also an increase in N-acetylglucosamine kinase (Shepherd et al., 1980). Likewise, encystment of *B. emersonii* zoospores which requires the formation of a chitin-containing CW also involves an increase in the enzyme activity (Frisa and Sonneborn, 1982; Maia, 1994).

Chemical analysis of the CW revealed the same levels of chitin in the two morphotypes of *S. schenckii* (Travassos and Lloyd, 1980). Nevertheless, since many macromolecules other than chitin depend on the functioning and regulation of the HBP, variations in the levels of GlcN-6-P synthase activity are expected to occur during growth and morphogenesis of the fungus. On this premise, a few years ago a project dealing with the purification and biochemical characterization of this enzyme from *S. schenckii* was started (Gonzalez-Ibarra et al., 2010) and later the interest moved on to explore other aspects related with the role of GlcN-6-P synthase in *Sporothrix* physiology and metabolism (Sánchez-López et al., 2015; Sánchez-López et al., 2019; Ortiz-Ramírez et al., 2021).

#### Native and recombinant GlcN-6-P synthase from *S. schenckii.* Purification and biochemical characterization

Based on protocols described to purify the enzyme from *C. albicans* (Milewski et al., 1999) and *E. coli* (Sachadyn et al., 2000), a procedure was developed to isolate the native enzyme to homogeneity from mycelia of *S. schenckii* (González-Ibarra et al., 2010). Briefly, the enzyme from the cytosol was consecutively treated with protamine sulfate, ammonium sulfate, polyethylene glycol and finally subjected to ion exchange and adsorption chromatographies. After the final step, enzyme recovery and purification were 46% and 106.2-fold, respectively. Analytical electrophoresis of the purified enzyme preparation revealed a single protein band of 79 kDa and an isoelectric point of 6.26 which is similar to that of 6.27 estimated for the enzyme from *A. niger* (Ram et al., 2004) and is well within the range of 5.56-6.42 calculated for the enzyme from *E. coli* (Dutka-Malen et al., 1988) and human (McKnight et al., 1992) cells. A native molecular weight (MW) of 350 ± 5 kDa was calculated by size exclusion chromatography thus corresponding to a homotetramer, in close agreement with the enzyme from other eukaryotes. This was the first report of a native GlcN-6-P synthase purified from a truly dimorphic fungus (González-Ibarra et al., 2010).

Biochemical characterization of the enzyme revealed a 2-fold higher affinity for fructose-6-P over L-glutamine considering the Km values of 1.12 and 2.2 mM, respectively (González-Ibarra et al., 2010). Other fructose-6-P/L-glutamine K_m_ values (all mM) are 0.007/0.26, 13.3/18.8, 2.9/0.56, 1.45/1.56 and 0.9/0.3 for the enzymes from human cells (Broschat et al., 2002), *Proteus mirabilis* (Cifuentes and Vicente, 1982), *N. crassa* (Endo et al., 1970), *C. albicans* (Milewski et al., 1999) and *S. cerevisiae* (Watzele and Tanner, 1989), respectively. These variations of K_m_ values may be due to structural differences around the catalytic sites involved in fructose-6-P and L-glutamine recognition among the different organisms.

Aside from the much larger MW than its counterpart from prokaryotes, another striking difference is that GlcN-6-P synthase from all eukaryotes so far examined is down regulated by UDP-N-GlcNAc (Milewski, 2002; Milewski et al., 2006). The observed effect of the aminosugar on the enzyme was a little surprising as the cytosolic enzyme was over 8-fold more sensitive than the pure enzyme as deduced from the IC_50_ values of 0.12 mM and 1.0 mM, respectively (González-Ibarra et al., 2010). The IC_50_ value of 0.12 mM is 5.6-5.8 lower than those estimated for *C. albicans* (Milewski et al., 1999) and *S. cerevisiae* (Watzele and Tanner, 1989) but 1.4-, 6.0- and 20-fold higher than in *N. crassa* (Endo et al., 1970), rat (Kornfeld, 1967) and human (Kikuchi and Tsuiki, 1976) liver, respectively. Seemingly, the presence of glucose-6-P but no other sugar phosphates in crude preparations of the enzyme increases the sensitivity to UDP-N-GlcNAc (Milewski et al., 1999; Milewski et al., 2006). In agreement with these observations, 10 mM glucose-6-P almost restored the sensitivity of pure GlcN-6-P synthase to 1 mM UDP-GlcNAc to the levels of crude or partially purified preparations of the enzyme. According to a study of the rat liver enzyme, glucose-6-P may be produced from fructose-6-P by a phosphoglucose isomerase present in the extracts (Miyagi and Tsuiki, 1974).

One report indicates that the sequence of the eukaryotic GlcN-6-P synthase contains several sites for phosphorylation by different kinases which is not held for the prokaryotic counterpart, thus marking another important difference between these organisms (Milewski, 2002). Regarding this issue, the relationship between enzyme activity, down regulation by UDP-N-GlcNAc and phosphorylation varies among fungi. For instance, the enzyme in zoospores but not in vegetative cells of *B. emersonii* is inhibited by UDP-N-GlcNAc and this effect increases after enzyme phosphorylation (Selitrennikoff et al., 1980). Interestingly, during zoospore germination, enzyme dephosphorylation results in a loss of sensitivity to the inhibitor to cope with a higher demand for chitin and other CW components (Frisa and Sonneborn, 1982; Etchebehere and Maia, 1989). In the same line, the phosphorylation of the enzyme in *C. albicans* by a cAMP-dependent protein kinase increased enzyme activity whereas inhibition by the aminosugar was poorly affected. Loss of sensitivity to the inhibitor occurred at the onset of yeast to mycelium transition by a mechanism involving a decrease in both glucose-6-P levels and enzyme phosphorylation (Milewski et al., 1999). In terms of this property, the enzyme from *S. schenckii* resembles that of *C. albicans*. Thus, enzyme activity increased 71% following incubation with a cAMP-protein kinase and ATP and addition of a kinase inhibitor significantly reduced activation. Enzyme phosphorylation did not significantly alter the enzyme sensitivity to inhibition by UDP-N-GlcNAc (González-Ibarra et al., 2010). Clearly, more studies will be required to elucidate the role of phosphorylation in the modulation of GlcN-6-P synthase and HBP.

#### Specific inhibitors of GlcN-6-P synthase and growth of *S. schenckii*

Much of the interest in GlcN-6-P synthase is based on its potential as a target for antimicrobial drugs. Since the use of anticapsin back in the 70’s (Neuss et al., 1970) and other products (Krynski et al., 1952; Rogers et al., 1965; Atsumi et al., 1975; Rapp et al., 1988), much work has been dedicated to the search for new inhibitors of GlcN-6-P synthase and fungal growth. Two of these compounds were tested in *S. schenckii*, namely FMDP [*N* ^3^-(4-metoxyfumaroyl)-L-2,3-diaminopropanoic acid] and ADMP (2-amino-2-deoxy-D-mannitol-6-phosphate) which are analogs of L-glutamine and the putative transition state intermediate, respectively. It was observed that FMDP was over 5-fold stronger inhibitor of GlcN-6-P synthase than ADMP as inferred from the IC_50_ values of 14.2 μM and 74 μM, respectively (González-Ibarra et al., 2010; **Figure 6**). These results contrast with previous findings indicating that ADMP as well as ADGP (2-amino-2-deoxy-D-glucitol-6-phosphate) are the strongest inhibitors of the enzyme (Janiak et al., 2003), suggesting a difference between the *S. schenckii* enzyme and that from other organisms.

The antifungal action of two FMDP-derived endothiopeptides Nva-FMDP and Lys-Nva-FMDP previously tested in *C. albicans* (Nowak-Jary and Andruszkiewicz, 2009) as well as FMDP, ADMP and two derivatives of ADPG like N-butanoil-ADPG and N-hexanoil-ADPG were tested on growth in microtiter plates containing YNB and RPMI 1640 culture media. In both media, FMDP, Nva-FMDP and Nva-Lys-FMDP did not reduce growth beyond a range of 11-39%. On the contrary, ADMP which was a poor enzyme inhibitor and the ADGP derivatives inhibited growth to over 90% (González-Ibarra et al., 2010; **Figure 7**). Amphotericin B, voriconazole and fluconazole were used as positive controls. Corresponding MIC values were 0.00039, 0.025 and 0.5 mg/ml in RPMI 1640 and 0.00156, 0.0125 and 0.05 mg/ml in YNB, respectively. The low antifungal action of FMDP and its derivatives may be due to the inability of *S. schenckii* to internalize these inhibitors due to the lack of a peptide permease or alternatively a fail to release the inhibitor inside the cell. Whatever the case, these findings provide further evidence for the role of the enzyme as a target for antifungals (González-Ibarra et al., 2010).

In order to purify the GlcN-6-P synthase from *S. schenckii*, the recombinant protein was obtained by cloning the *GFA1* gene in the expression plasmid pYEX-BX obtaining the pYEX-SsGFA1 construction which was used to transform *S. cerevisiae* 8A and the recombinant strain was labeled as 8A-pYEX-SsGFA1. The enzyme was purified and some of its properties were compared with those of the native enzyme (Sánchez-López et al., 2015). Bioinformatic analysis of GlcN-6-P synthase of *S. schenckii* revealed a MW of 78.4 kDa and an isoelectric point of 6.1, which are consistent with the experimental values determined earlier for the native enzyme (González-Ibarra et al., 2010). The protein lacks potential sites for O- and N-glycosylation, it does not carry a signal peptide and contains several potential sites for phosphorylation. These properties are similar to those previously reported for the enzyme from other eukaryotes.

The functionality of GlcN-6-P synthase in the pYEX-SsGFA1 construction was assessed in the strain 8A of *S. cerevisiae* (A.J.P. Brown, Aberdeen, UK). This strain was auxotrophic for adenine, leucine, histidine and D-glucosamine and failed to grow on amino acid-free YNB lacking Leu or Leu plus GlcN, but it was able to do so upon transformation with the pYEX-SsGFA1 construction (8A-pYEX-SsGFA1), thereby indicating the presence in the mutant of a functional GlcN-6-P synthase. Pure recombinant GlcN-6-P was biochemically evaluated in order to determine its stability, substrate effect and susceptibility to inhibitors. Comparison of results obtained with the recombinant enzyme with those of the native protein, indicated that the recombinant enzyme has a greater stability, less susceptibility to UDP-GlcNAc, and the same susceptibility to inhibitors (Sánchez-López et al., 2015). These results are consistent with those reported in other eukaryotes.

### CW changes induced by environmental stress

**Figure 2:**
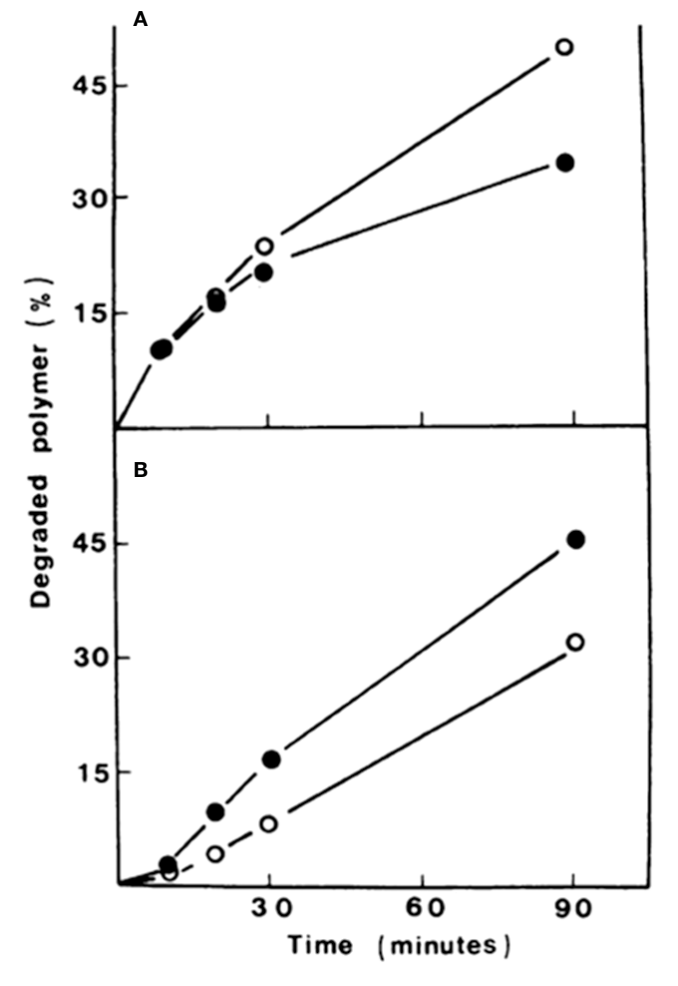
Degradation rates of β(1-3)-glucan **(A)** and chitin **(B)** synthesized *in vitro* in the presence (closed circles) or absence (open circles) of Calcofluor. The initial amount of polysaccharide to be degraded was ca. 260 nmol (ca. 300,000 cpm). Modified from Roncero and Durán (1985).

Dyes like Congo Red (CR), Calcofluor White (CFW) and other compounds that interact with CW constituents, particularly crystalline polymers, have been reported to alter the CW causing severe changes in its appearance and cell morphology (Bartnicki-Garcia et al., 1994; Bermejo et al., 2010). In this context, it has been observed that these perturbing agents inhibit β-glucan and chitin synthases in several fungi (Selitrennikoff, 1984; Roncero and Duran, 1985; Vermeulen and Wessels, 1986). *In vivo*, CR and CFW induce an increase in the synthesis of chitin and glucan in *Saprolegnia monoica* (Fèvre et al., 1990; Nodet et al., 1990a; Nodet et al., 1990b) and *Geotrichum lactis* (Roncero and Duran, 1985). In this context, it was observed that CFW was able to revert protoplasts from *G. lactis* and *S. cerevisiae* by increasing the synthesis of both alkali-insoluble β(1,3)-glucan and chitin (**Table 1**). However, it was intriguing to observe that neither CFW, CR, nor WGA were able to activate chitin synthesis *in vitro*. In contrast, CFW caused the degradation of *in vitro*- synthesized polysaccharides (**Figure 2**) (Roncero and Duran, 1985).

**Table 1:** Effect of Calcofluor on the synthesis of cell wall polysaccharides in reverting protoplasts from *G. lactis* and *S. cerevisiae*.

		(cpm incorporated per fraction/ total cpm incorporated) x 10^2b^			
Organism	Polysaccharide	Control cultures		Calcofluor treated cultures	
		A	B	A	B
*G. lactis*	Alkali-insoluble β(1,3)-glucan	0.7	0.7	5	13
	Chitin	1.1	1.3	11.7	42
*S. cerevisiae*	Alkali-insoluble β(1,3)-glucan	2	9	3.7	8.5
	Chitin	1.3	4.3	4.6	10.3

### Compensatory responses of *S. schenckii* to CW damage

As mentioned before, the dynamic nature of CW adapts to changes in growth conditions and external factors and can be remodeled/repaired in a way to participate in morphogenetic processes such as mating, sporulation, and morphological transition (Orlean, 1997; Smits et al., 1999; Molina et al., 2000). Adaptation has been demonstrated by cell compensatory mechanisms that allow the CW to modify its chemical composition in response to environmental stress (Kapteyn et al., 1997; Popolo et al., 2001; Klis et al., 2002). Most of what we presently know about the molecular basis of compensatory responses to wall damage comes from studies in *S. cerevisiae* where responses to maintain CW integrity were analyzed in five yeast mutants affected in wall elaboration. This analysis revealed that perturbation induced by these mutations triggered three complex regulatory systems and that 33 out of the 290 differentially expressed genes were involved in the remodeling/repair of the wall including some related to chitin synthesis such as *GFA*1, *AGM*1 and *CHS3* (Lagorce et al., 2003). Moreover, treatment of *S. cerevisiae* with CR and Zymolyase elicited the expression of a number of genes involved in wall synthesis and degradation (Garciía et al., 2004), some of which were regulated by a phosphorylation cascade involving protein kinase C (Arellano et al., 1999; Heinisch et al., 1999; Katayama et al., 1999; Calonge et al., 2000; Valdivia and Schekman, 2003; Ichinomiya et al., 2007).

#### Influence of CR on GlcN-6-P synthase, growth and morphology of *S. schenckii*

**Figure 3:**
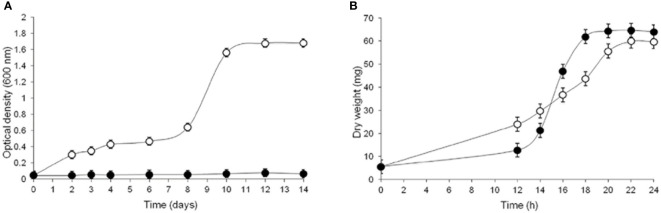
Influence of CR on growth of both morphotypes of *S. schenckii*. CN were inoculated and allowed to propagate in YPG medium in conditions set to produce yeast **(A)** or mycelial **(B)** cells in the absence (open circles) and presence (closed circles) of 15 μM **(A)** and 150 μM **(B)** CR and growth was evaluated after the indicated times. Reproduced from Sánchez-López et al., 2019, with permission from Springer Nature, License Number 501734282 (May 24, 2022).

To explore the compensatory responses of *S. schenckii* to CW damage, the effects of CR were investigated in terms of cell viability, morphology and GlcN-6-P synthase in both yeast and mycelium morphotypes propagated in YPG medium (Bartnicki-García and Nickerson, 1962) either from conidia (CN) or the corresponding vegetative cells (Sánchez-López et al., 2019). A first striking observation was the failure of CN to germinate when incubated under conditions to produce yeast cells in the presence of 15 μM CR. However, in control cultures yeast cells grew slowly up to 8 days of incubation; thereafter, a rapid increase was observed showing a maximum growth after 12 days (**Figure 3A**). In contrast, in culture conditions to produce mycelium, CN germinated giving rise to filamentous cells even in the presence of a 10-fold higher concentration of CR. Under these conditions, a slight inhibition of the mycelial morphotype was observed during the first 12-14 h but then mycelial cells grew normally outgrowing control cells (**Figure 3B**) (Sánchez-López et al., 2019).

**Figure 4:**
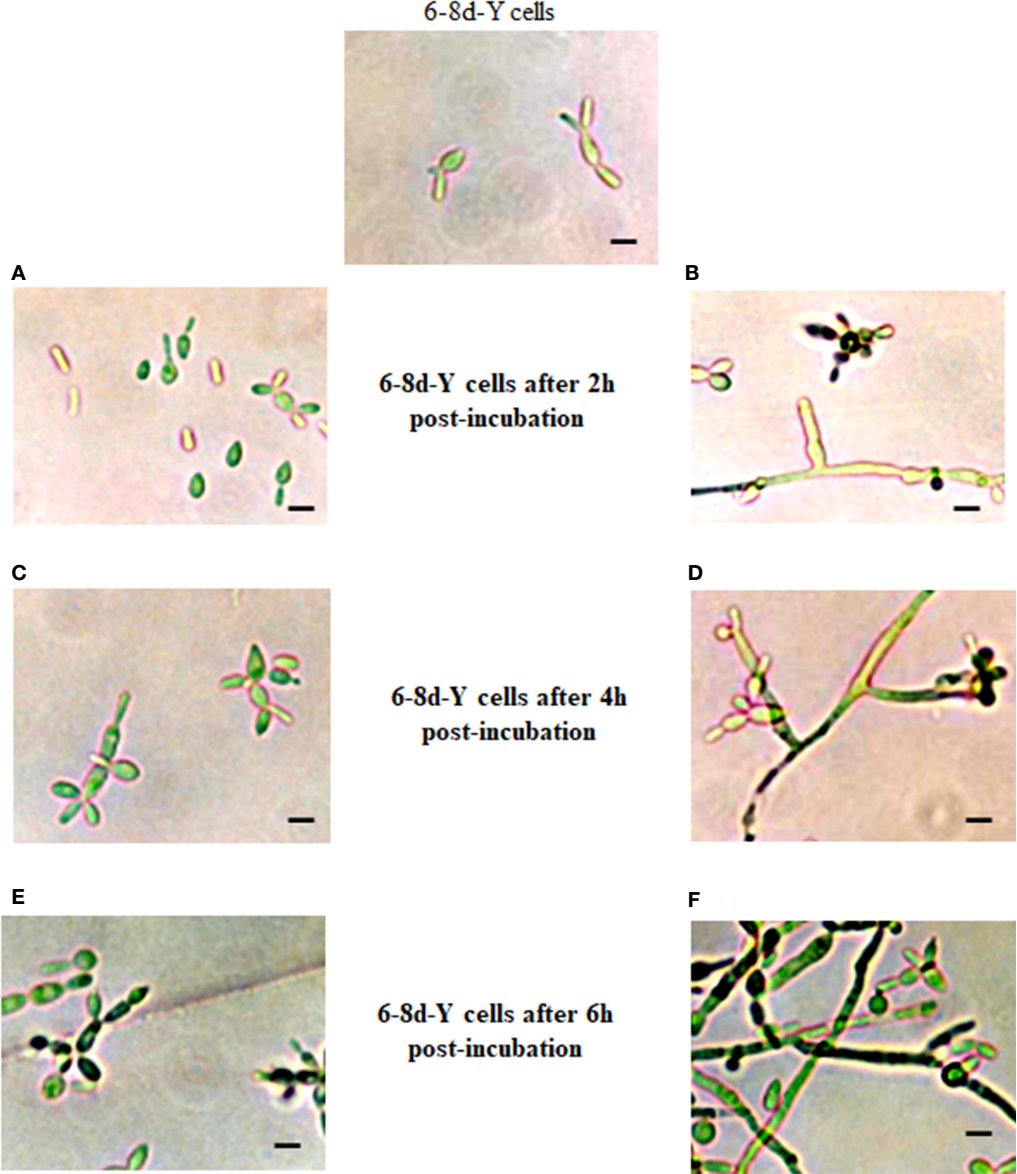
Influence of CR on growth and morphological development of yeast cells. A culture of yeast cells grown for 6-8 days in the absence of CR was divided into two equal portions that were post-incubated in the same conditions for 2 **(A, B)**, 4 **(C, D)** and 6 **(E, F)** h in the absence **(A, C, E)** and presence **(B, D, F)** of 15 μM CR. Cell morphology was inspected after the indicated times by OM. Bar, 5 μm. Reproduced from Sánchez-López et al., 2019, with permission from Springer Nature, License Number 501734282 (May 24, 2022).

Results described in **Figure 3** led authors to test CR on cells pre-grown in the absence of CR and then post-incubate the produced vegetative cells in the presence of the dye. To this purpose, CN were incubated in the absence of CR for either 6-8 days and 16 h in conditions adjusted to obtain the yeast and mycelium morphotypes, respectively. These cultures were correspondingly named as 6-8d-Y and 16h-M cells. After these times, CR-free cultures were divided into two equal parts: one half of 6-8d-Y and 16h-M cells received 15 μM and 150 μM CR, respectively, while the other portions were left as controls. Cultures were then post-incubated for 2,4 and 6 h (6-8d-Y cells) and 5 and 10 h (16h-M cells) and observed by optical (OM) and transmission electron (TEM) microscopies. Post-incubation of 6-8d-Y cells in the presence of CR induced a rapid differentiation of yeasts into the mycelium morphotype (compare **Figures 4A, B**) and the effect was more evident after 4 and 6 h where branched and septate mycelia and CN were clearly visible (**Figures 4D, F**). In the absence of CR, the culture exhibited a regular, yeast growth profile (**Figures 4C, E**). This was an unexpected result since it occurred in conditions set to obtain the yeast morphotype suggesting that *S. schenckii* avoids the harmful effect of the dye by switching to an insensitive morphology even under conditions not propitious for mycelial growth. This morphological response is not simple to explain in simple terms though it is conceivable that growth yeast development generates a CW structure more exposed to the agent than the mycelial morphotype (Sánchez-López et al., 2019).

In the case of mycelium, the effect of CR was limited to structural changes similar to those observed in other fungi (Vannini et al., 1983; Fèvre et al., 1990; Nodet et al., 1990a). Accordingly, following incubation of 16h-M cells for 5 h in the absence of CR, OM showed a regular, apical growing hyphae with a normal polarity and inspection of a cross section by TEM showed the presence of vesicles, a regular CW and nuclei. In contrast, post-incubation for 5 h in the presence of CR gave rise to hyphae with swollen ends and a clear loss of polarity, with a width 2- or 3-fold larger than the normal hyphae and a red-colored cell surface. TEM, on the other hand, revealed irregularly shaped, distorted cells surrounded by an electron-dense CW and accumulated intracellular pigmented bodies of variable size. Whether this pigment was CR was not investigated further. When incubation of 16h-M cells was extended to 10 h without the dye, cells showing a typical hyphal tip with a regular septum, apical vesicles, nuclei and a normal CW were observed. In contrast, post-incubation

with CR produced red-stained surface filamentous cells and internal vesicles with branched globose structures. Observation of ultra-thin sections by TEM showed an electron-dense CW and an aberrant, atypical fried egg-like cell appearance (Sánchez-López et al., 2019; **Figure 2**).

Fungi respond to CW perturbations by a global transcriptional mechanism that involves the differential expression of several genes related to wall elaboration. One of these is *GFA1* encoding GlcN-6-P synthase for activation of chitin synthesis (Jung and Levin, 1999; Lagorce et al., 2003; García et al., 2004). In this regard, it was considered relevant to explore the effect of CR on the activity of the enzyme that regulates the levels of UDP-N-GlcNAc. In a first experiment, it was observed that 16h-M cells exhibited a low, basal enzyme activity along the time of incubation in the absence of CR. In its presence, however, enzyme activity started to increase reaching an optimum after 3 h that corresponded to a 7-fold increase over the mock cells, followed by a decrease to basal levels after 6 h of incubation (Sánchez-López et al., 2019; **Figure 3**). This result supports previous reports that demonstrate that activation of chitin synthesis in yeast wall mutants is concomitant with a proportional increase in GlcN-6-P synthase (Lagorce et al., 2002) and agrees with the observation that activation of GlcN-6-P synthase in *A. niger* results in an increased deposition of chitin in the wall (Ram et al., 2004).

#### Influence of CR and CFW on viability and growth of mycelium and yeast morphotypes of *S. globosa*

In fungi and other organisms, damage of the cell surface by external agents triggers compensatory responses aimed to maintain the integrity and functionality of the CW. Evidence for these observations were contributed to a report on *S. schenckii* whose CW was altered by the binding of CR and the potential involvement of GlcN-6-P synthase in these responses (Sánchez-López et al., 2019). This is a critical enzyme for CW biogenesis that has been purified and characterized in its native and recombinant forms from *S. schenckii* and their properties compared with their counterparts in other organisms (González-Ibarra et al., 2010; Sánchez-López et al., 2015). To get a further insight into the fungal compensatory responses, with emphasis on the role of GlcN-6-P synthase, these studies were extended to *S. globosa*, another pathogenic member of the *Sporothrix* genus that exhibits several differences with other species of the same genus and included CFW in addition to CR (Ortiz-Ramírez et al., 2021).

Influence of dyes on cell viability, either individually or combined, was assayed at concentrations of 0-300 μM and 0-2% for CR and CFW, respectively, and the same concentrations were maintained in the mixture. Based on the results, 150 μM CR and 1% CFW, either individually or mixed, were chosen for further studies with the mycelium. Values for the yeast morphotype were 15 μM CR and 1% CFW (Ortiz-Ramírez et al., 2021). It should be pointed out that in experiments carried out to determine the effect of the dyes on both morphotypes, mycelial cells were propagated using CN as inoculum, whereas for the yeast morphotype, an inoculum of vegetative cells was first prepared by growing CN for 4-6 days under conditions to obtain this morphology since it has been demonstrated that CR inhibits CN germination (Sánchez-López et al., 2019). Prior to propagation, the inoculum was filtered to get rid of some mycelia that might have been formed during pre-incubation.

**Figure 5:**
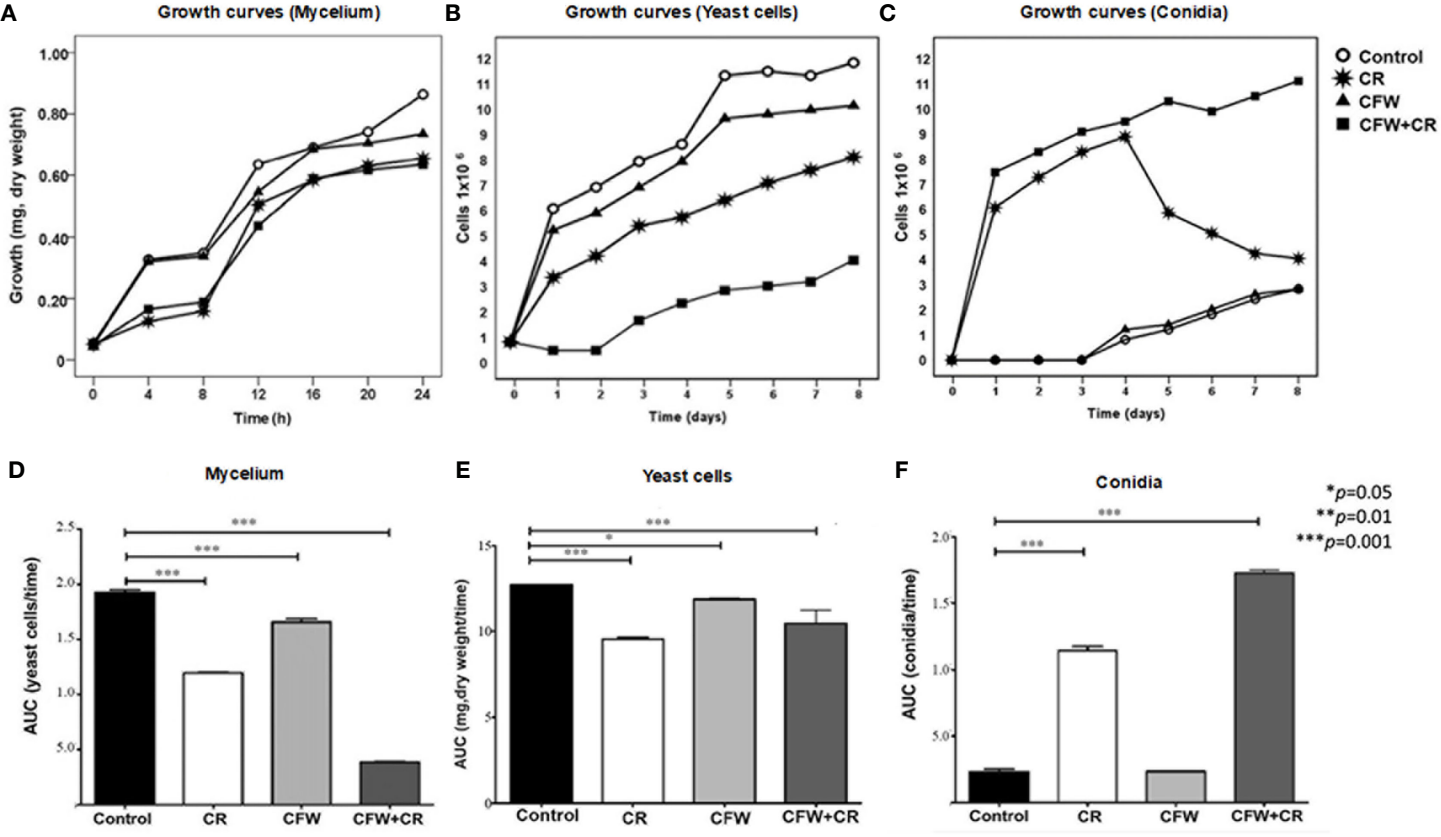
Influence of CR and CFW on growth of *S. globosa*. In **(A)** growth of mycelial cells was determined after the indicated times of incubation in the absence and presence of 150 μM CR or 1% CFW either alone or in combination. Growth of the yeast morphotype was assessed after the indicated times by counting either the yeast cells **(B)** or CN **(E)**. The statistics analyses presented in **(B, D, F)** were calculated from the values obtained from the area under the curve (AUC) for each growth condition. Reproduced from Ortiz-Ramirez et al., 2021, with permission from Springer Nature, License Number 5315470121400 (May 24, 2022).

As depicted in **Figure 5A**, CFW did not significantly affect mycelial growth whereas CR, either alone or mixed with CFW, reduced growth to about 50% when incubation was extended to 4 and 8 h. Thereafter, fungal growth was partially recovered without reaching that of mock cells. Different results were obtained with the yeast morphotype whose growth was reduced to a significant level after 1 day of exposure to CFW and this effect was maintained up to the end of the experimental period. CR, either alone or combined with CFW, reduced growth to 50% and 92%, respectively, after one day of incubation and the inhibitory effect was maintained along the experimental time (**Figure 5C**). It has been proposed that growth inhibition by these stressants is due to the rigidness imposed by the binding to the fungal surface and the resulting inability of the cell to appropriately re-structure the CW (Ram and Klis, 2006). Recently, the notion was advanced that the compensatory responses of *S. cerevisiae* to cell surface stress by CR, Zymolyase and SDS are mediated by a CW integrity (CWI) pathway through Mid2, a membrane-resident mechanosensor, that transduces the signal to a protein phosphorylation pathway and the transcription factor R1m1, giving rise to a strong inhibition of growth (Jiménez-Gutiérrez et al., 2020b; Jiménez-Gutiérrez et al., 2020a). As a matter of fact, the role of a PKC has been reported in *S. schenckii* (Aquino-Piñero and Rodríguez-del-Valle, 2002; Ichinomiya et al., 2007) thus supporting the hypothesis that a comparable mechanism may function in *S. globosa*.

Light microscopy of yeast cultures exposed to CR, or the mixture revealed the presence of mycelial cells and abundant CN. These prompted the authors to measure growth by counting the number of CN. Results revealed that the growth curves obtained under the different culture conditions were almost the inverse of those observed in **Figure 5C**. Accordingly, control cultures and those containing CFW did not produce CN after 3 days of incubation; thereafter, the CN count started to increase slowly up to the end of the incubation period. In a notorious contrast, cultures containing CR and the mixture produced abundant CN derived from conidiogenic filamentous cells formed from yeast cells (**Figure 5E**). These CN were morphological indistinguishable from those generated from mycelia grown under normal conditions. CR-induced morphological transition of yeast to mycelial cells confirmed previous results in *S. schenckii* (Sánchez-López et al., 2019) and most likely corresponds to a mechanism that permits the fungus to evade the noxious effect of the dye. Statistics analysis of data shown in **Figures 5A, C, E** are presented in **Figures 5B, D, F**, respectively.

#### Effect of CW-interacting dyes on morphology of *S. globosa*

**Figure 6:**
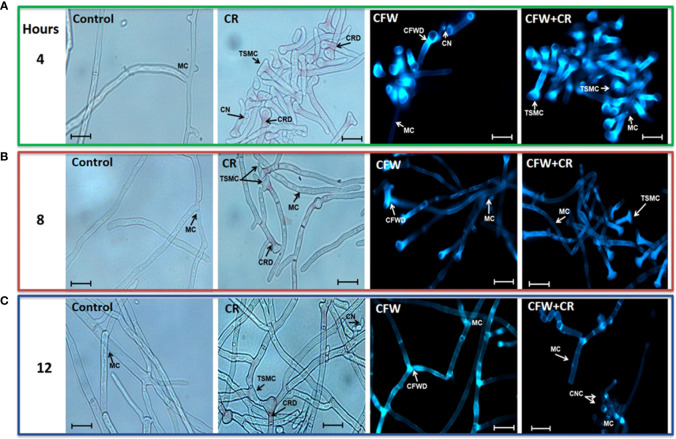
Mycelial morphology of *S. globosa* in response to CR and CFW. The mycelial morphotype was propagated in the absence or presence of CR, CFW or the mixture and after the indicated times, cultures were inspected by brightfield and epifluorescence microscopies to register the cell morphology. CN, conidia; MC, mycelial cell; TSMC, T-shaped mycelial cell; CRD, CR deposit; CFWD, CFW deposit; CNC, CN clusters. Bar, 20 μm. Reproduced from Ortiz-Ramirez et al., 2021, with permission from Springer Nature, License Number 5315470121400 (May 24, 2022).

Morphology of mycelium and yeast morphotypes incubated under different conditions was observed by brightfield (BFM) and epifluorescence (EFM) microscopies. For mycelium, fresh CN were inoculated in YPG medium with or without 150 μM CR or 1% CFW, either alone or in combination. Cultures were incubated for a period of 24 h and cell morphology was inspected every 4 h. Essentially the same protocol was set for the yeast morphotype except that yeast vegetative cells were used as inoculum and 15 μM CR and 1% CFW were used. It should be pointed out that in this case the incubation period was extended from to 2, 4 and 6 h used earlier (Sánchez-López et al., 2019) to 1, 4 and 6 days with the aim to investigate whether there was a time of return of the filamentous cells formed from yeast cells incubated with the dyes (**Figure 6**). Though fresh CN were incubated for 24 h and inspected every 4 h, only images obtained after 4, 8 and 12 h of incubation are shown since these were the times where the most notorious effects were observed in several experiments. After 4 h, control cultures contained hyphae of regular morphology while in those containing CR, T-shaped cells with a clear loss of polarity, globose structures and red-pigmented deposits, presumably of CR, were noticed. CFW by itself or combined with CR accumulated all over the cell surface (**Figure 6A**). After 8 h, CR deposits started to disappear and those remaining were observed mainly on the branching points. Some septa were occasionally seen. On its part, CWF alone or combined with CR stained the cell surface with a preference for the globose apex (**Figure 6B**). Essentially the same observations were made after 12 h where some CN clusters started to appear and CFW deposited mainly at the branching points (**Figure 6C**) (Ortiz-Ramírez et al., 2021).

**Figure 7:**
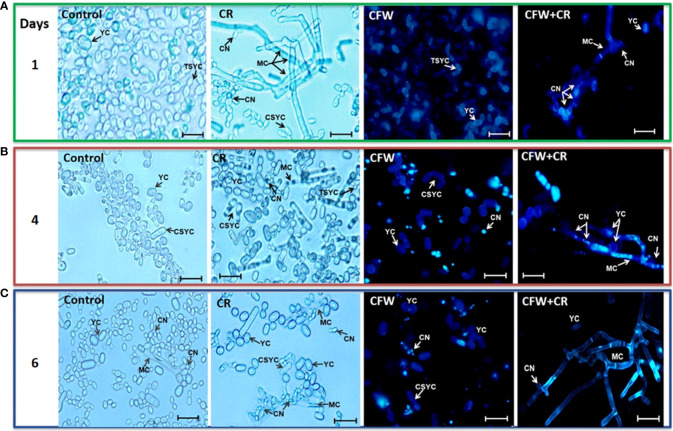
Yeast morphology of *S. globosa* in response to CR and CFW. The yeast morphotype was propagated in the absence or presence of CR o CFW and after the indicated times, cultures were inspected by brightfield and epifluorescence microscopies to register the cell morphology. CN, conidia; YC, yeast cell; MC, mycelial cell; TSYC, tear-shaped yeast cell; CSYC, cigar-shaped yeast cell. Bar, 20 μm. Reproduced from Ortiz-Ramirez et al., 2021 with permission from Springer Nature, License Number 5315470121400 (May 24, 2022).

On the other hand, images captured after 1, 4 and 6 days of incubation of yeast cells with the dyes confirmed the yeast-to-mycelium transition described before (**Figure 7**). Accordingly, after 24 h of incubation, CR triggered a remarkable morphological shift of yeasts to conidiogenic mycelia and CN whereas only tear-shaped yeast cells were observed in mock cultures (**Figure 7A**). Alone, CFW stained all over the yeast cell surface and failed to induce the morphological change caused by CR. After 4 days, CR-containing cultures exhibited a complex, heterogenous population of yeast and mycelium cells in what it appeared to be an intermediate phase of the regression process of mycelia to yeasts, and an ensuing decrease of CN. With CFW, some fluorescent cells were observed (**Figure 7B**). After 6 days, filamentous cells induced by CR returned back to yeast cells with a significant decrease of mycelia as compared with results obtained after one day of incubation (**Figure 7C**). Most probably, regressive transition occurred after degradation of CR to an innocuous product. An intriguing observation was that mycelia formed in the presence of combined CR and CFW failed to return to yeast cells (**Figure 7C**). A hypothesis for this behavior is proposed below.

Two alternatives to explain the morphological transition induced by CR were advanced by Ortiz-Ramírez et al. (2021). On one hand, it is conceivable that response to the dye to the aggression of either the CW or the plasma membrane involves a membrane-associated sensor functionally equivalent to one of those found in *S. cerevisieae*, which activates a signal pathway that ultimately induces the morphological change, as has been proposed for other responses to extracellular factors (Elhasi and Blomberg, 2019). This signal transduction route is known as PKC1-SLT1 cascade (Gualtieri et al., 2004). An evidence in support of this proposal is that one of these sensors has been reported in *S. schenckii* and *S. brasiliensis* (Teixeira et al., 2015) though not yet in *S. globosa*. In a second alternative, mechanosensitive ion channels responsible for responses to external stimuli in many cell types (Kumamoto, 2008) may also occur in *S.globosa* and, after opening, allow the entrance of CR into the cell where it outputs the signal pathway for the morphological transition. It should be noticed that the dye has not been observed in mycelia produced from yeasts most likely because the low amount of CR (15 μM) used for the yeast morphotype as compared with that of the mycelium morphotype (150 μM). Whatever the case, heretofore this is first report of the morphological transition induced by CW damage of a truly dimorphic fungus.

A reflection derived from these observations is that selective growth conditions to obtain the yeast morphotype cease to be restrictive for mycelial growth when the organism confronts the need to survive in an adverse milieu.

#### GlcN-6-P synthase as a compensatory response to wall damage

A major goal of studies dealing with compensatory responses to cell surface stress was to evaluate the involvement of GlcN-6-P synthase. Supporting reports of this role have been provided by the activation of chitin synthesis in wall mutants of *S. cerevisiae* which correlates with an increase in GlcN-6-P synthase activity (Lagorce et al., 2002; Ram et al., 2004) and, vice versa, that activation of GlcN-6-P synthase in *Aspergillus niger* results in an augmented deposition of chitin in the CW (Ram et al., 2004). In the same line, other authors reported that incubation of mycelial cells of *S. schenckii* with CR induced a significant increase in enzyme activity (Sánchez-López et al., 2019).

**Figure 8:**
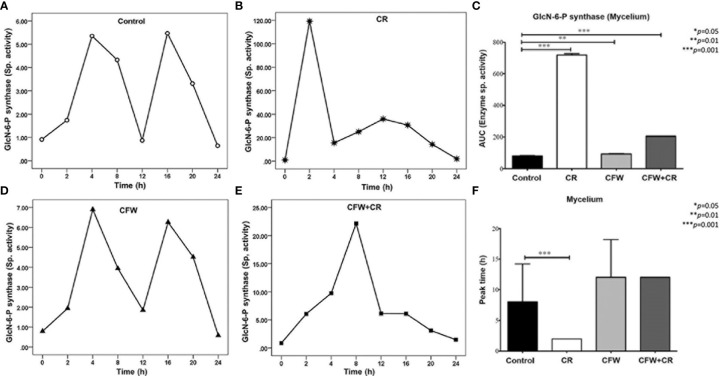
Responses of GlcN-6-P synthase of the mycelium of *S. globosa* to CW damage induced by CR and CFW. Mycelia was propagated in the absence and the presence of the dyes either alone or in combination. After the indicated times, cells were processed to measure enzyme activity. Control culture and those containing CR, CFW and the mixture are illustrated in Figures **(A) (B) (C)** and **(D)** respectively. Statistics analyses were carried out by calculating the area under the curve (AUC) **(E)** and the time required for the enzyme to reach its maximum activity **(F)**. Reproduced from Ortiz-Ramirez et al., 2021, with permission from Springer Nature, License Number 5315470121400 (May 24, 2022).

Further analysis of the enzyme behavior in response to the dyes revealed that incubation of the mycelium morphotype of *S. globosa* in the absence of CR gave rise to two peaks of similar activity after 4 and 16 h (**Figure 8A**). In the presence of CR, however, two peaks appeared 2-4 h earlier and exhibited a 24- and 7-fold higher activity as compared with the corresponding peaks in control cells (**Figure 8B**). It was proposed that the second peak in CR-treated mycelia may correspond to that observed earlier under similar conditions (Sánchez-López et al., 2019). With CFW, enzyme peaks did not differ much in activity and the time of appearance with respect to those observed in mock cells (**Figure 8C**). With the mixture, only one peak was observed after 8 h showing a 4.5-fold higher activity with respect to mock culture (**Figure 8D**). Results obtained with the mixture matched with the inability of mycelium cells to transit back to yeasts cells in the presence of both dyes, as noticed in **Figure 7C**, and strengthen the idea that CFW interferes with the effect of CR by a mechanism yet to be elucidated. Statistics analysis of all culture conditions are presented in **Figures 8E and F**. When values obtained with CR and the mixture were compared with control cells, the analysis of AUC revealed a highly significant difference as compared with CFW (**Figure 8E**). Moreover, results indicated that in the presence of CR, the enzyme reached an overpeak after 2 h whereas it required 8 h to do so in control cells (**Figure 8F**).

**Figure 9:**
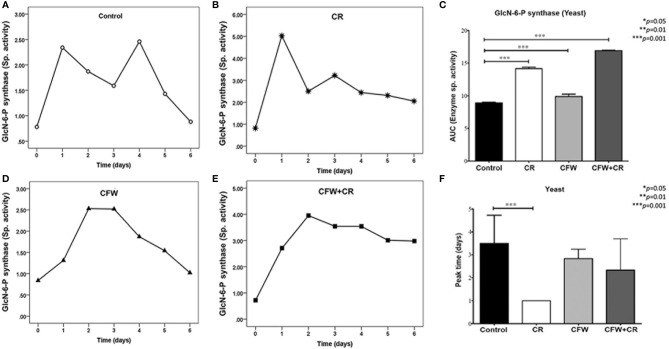
Responses of GlcN-6-P synthase from yeast cells of *S. globosa* to CW damage induced by CR and CFW. Yeast cells were propagated in the absence and the presence of the dyes either alone or in combination. After the indicated times, cells were processed to measure enzyme activity. Control culture and those containing CR, CFW and the mixture are illustrated in Figures **(A–D)** respectively. Statistics analyses were carried out by calculating the area under the curve (AUC) **(E)** and the time required for the enzyme to reach its maximum activity **(F)**. Reproduced from Ortiz-Ramirez et al., 2021 with permission from Springer Nature, License Number 5315470121400 (May 24, 2022).

Responses of yeast cells to dyes in terms of GlcN-6-P synthase activity differed from those of mycelial cells and were more complex. Thus, two peaks of similar activity were seen in control cultures after 1 and 4 days of incubation but significantly lower than those of mycelial cells (**Figure 9A**). In the presence of CR, the first peak augmented about 2.6-fold while the second one, which appear 1 day earlier, did not significantly increase over the corresponding control peak (**Figure 9B**). In response to CFW, yeasts expressed one peak after 2-3 days with a comparable activity to control cells (**Figure 9C**) while in the presence of the mixture, a peak of maximum activity was obtained after 2 days which slowly decreased. Again, activity was lower than that obtained with CR alone (**Figure 9D**). Statistics analysis of data obtained with yeast cells incubated under all conditions are presented in **Figures 9E, F**. A highly significant difference was observed when values obtained with CFW, CR and the mixture were compared with mock cells (**Figure 9E**). Overpeak values were 3.5, 1.0, 2.8 and 2.3 days in control cells and those exposed to CR, CFW and the mixture, respectively (**Figure 9F**).

It is well documented that CR and CFW bind to yeast CW chitin interfering with proper wall synthesis (Sanz et al., 2018). As mentioned above, CR is sensed mainly through Mid2 triggering the activation of cell responses to remodelate and stabilize the yeast CW. An ortholog of Mid2 has not yet been identified in *S. globosa* probably because not all genes are still annotated in this species. Other sensors in the yeast membrane include Wsc1, Wsc2, Wsc3 and Mtl1 which function to detect other environmental aggressors of the cell surface. It is conceivable that a membrane protein functionally similar to one of these mechanosensors plays the role of Mid2 in *Sporothrix* thereby sensing CR and causing the cell responses described here. An exhaustive search of the literature failed to find the sensor of CFW. However, as both dyes interact with chitin, it seems reasonable to infer that CR and CFW might share the same mechanosensor in *Sporothrix*. If this holds true, we hypothesize that CFW competes with CR resulting in a decrease or abolishment of CR effects. For instance, CFW by itself did not activate GlcN-6-phosphate synthase (**Figure 8**) nor promoted the conversion of mycelium back to yeast cells as CR did (**Figure 7**), just to mention a few effects.

The lower enzyme activity detected in yeast with respect to mycelial cells as well as the presence of two peaks in both control cells and some of the other culture conditions, are not easy to explain in simple terms. A plausible explanation is that hyphal cells may require more chitin and consequently more UDP-N-GlcNAc to form the diverse structures associated with mycelial growth such as long, septate and branched conidiogenic hyphae and CN, which are not seen in yeast cells. On the other hand, a possible explanation for the expression of two peaks of activity is that early during incubation, fungal growth and hence wall synthesis depend on a pool of aminosugar precursors that once consumed by the biosynthesis of wall polymers and other products, it has to be replenished by the enzyme activity associated to the first peak. Later, after incubation continues, limitation of aminosugar and other nutrients may act as metabolic signals that ultimately activate genes connected with wall synthesis giving rise to the second peak.

## Conclusions and future perspectives

Detailed reports discussed in this review on the compensatory responses of two species of the pathogenic clade of *Sporothrix* genus and other fungi to CW damage by the binding of CR and CFW, strengthen the generally accepted notion that the structure and composition of the fungal outermost layer is critical to maintain the cell functionality and survival. These responses include a number of complex processes whose coordinate integration contribute to repair and remodel the CW in a way that it continues supporting all the known functions associated to its presence in the cell. Among many other responses, stand out the rapid and transient conversion to a less sensitive or resistant morphological phase even under unfavorable conditions, as it is the case to the response to CR and, to a lower extent to CFW, reported for the first time in this review. Also, the observed variation in the activity of GlcN-6-P synthase, a crucial enzyme for CW biosynthesis due to its rate-limiting role in hexosamine biosynthesis. Major perspectives should include further research to explain some observations made in these studies, such as: i) Why do CN fail to germinate in the presence of CR under conditions to obtain the yeast morphotype but they do germinate under conditions to propagate the mycelium? ii) What mechanisms use yeast cells to convert and transiently grow as mycelial cells in the presence of CR under culture conditions presumptively not formulated for yeast growth? iii) When used together, how does CFW impact the effect of CR, for instance, the switch of mycelial cells back to yeast cells? iv) Is there a pool of the glucosamine-6-P synthase transcript which is translated in response to metabolic needs or, alternatively, there is a pool of inactive enzyme that is activated by posttranslational modification when needed? v) How CR and to a lower extent CFW trigger a signaling pathway in *S. globosa* that ultimately activates the *GFA1* and certainly other genes involved in wall biogenesis?

## Author contributions

EL-R and JO-R wrote and edit this manuscript and MC-C contributed to these actions. All authors contributed equally to the writing of this review and approved its submission.

## Funding

This investigation was done with the financial support granted to MC-C by Project No. CF2019-39216 from the National Council of Science and Technology (CONACyT), México, and Institutional grants UGTO-017/2022 and UGTO-07/2022 from University of Guanajuato, México, through the Direction of Support for Research and Posgraduate granted to MC-C and EL-R, respectively. JO-R received a Research Assistant scholarship from CONACyT.

## Acknowledgments

The authors acknowledge Springer Nature and Oxford University Press for permissions to reproduce/modify published material.

## Conflict of interest

The authors declare that the research was conducted in the absence of any commercial or financial relationships that could be construed as a potential conflict of interest.

## Publisher’s note

All claims expressed in this article are solely those of the authors and do not necessarily represent those of their affiliated organizations, or those of the publisher, the editors and the reviewers. Any product that may be evaluated in this article, or claim that may be made by its manufacturer, is not guaranteed or endorsed by the publisher.

## References

[B1] Bartnicki-García S. Nickerson W. J. (1962) Nutrition, growth, and morphogenesis of *Mucor rouxii* . J. Bacteriol. 84, 841–858. doi: 10.1128/jb.84.4.841-858.1962 13969720 PMC277967

[B2] Al-Tawfiq J. A. Wools K. K. (1998). Disseminated sporotrichosis and *Sporothrix schenckii* fungemia as the initial presentation of human immunodeficiency virus infection. Clin. Infect. Dis. 26, 1403–1406. doi: 10.1086/516356 9636870

[B3] Aquino-Piñero E. Rodríguez-del-Valle N. (2002). Characterization of a protein kinase c gene in *Sporothrix schenckii* and its expression during the yeast-to-mycelium transition. Med. Mycol. 40, 185–199. doi: 10.1080/mmy.40.2.185.199 12058732

[B4] Arellano M. Valdivieso M. H. Calonge T. M. Coll P. M. Duran A. Perez P. (1999). *Schizosaccharomyces pombe* protein kinase c homologues, pck1p and pck2p, are targets of rho1p and rho2p and differentially regulate cell integrity. J. Cell Sci. 112, 3569–3578. doi: 10.1242/jcs.112.20.3569 10504305

[B5] Arroyo J. Farkaš V. Sanz A. B. Cabib E. (2016). Strengthening the fungal cell wall through chitin–glucan cross-links: effects on morphogenesis and cell integrity. Cell. Microbiol. 18, 1239–1250. doi: 10.1111/cmi.12615 27185288

[B6] Assrir N. Richez C. Durand P. Guittet E. Badet B. Lescop E.. (2014). Mapping the UDP-n-acetylglucosamine regulatory site of human glucosamine-6P synthase by saturation-transfer difference NMR and site-directed mutagenesis. Biochimie 97, 39–48. doi: 10.1016/j.biochi.2013.09.011 24075873

[B7] Atsumi K. Oiwa R. Omura S. (1975). Production of bacillin by *Bacillus* sp. strain no. KM-208 and its identity with tetaine (bacilysin). J. Antibiot. 28, 77–78. doi: 10.7164/antibiotics.28.77 1126868

[B8] Badet B. Vermoote P. Haumont P. Y. Lederer F. Le Goffic F. (1987). Glucosamine synthetase from *Escherichia coli*: purification, properties, and glutamine-utilizing site location. Biochemistry 26, 1940–1948. doi: 10.1021/bi00381a023 3297136

[B9] Badet B. Vermoote P. Le Goffic F. (1988). Glucosamine synthetase from *Escherichia coli*: kinetic mechanism and inhibition by N3-fumaroyl-L-2, 3-diaminopropionic derivatives. Biochemistry 27, 2282–2287. doi: 10.1021/bi00407a006 3132968

[B10] Barreteau H. Kovač A. Boniface A. Sova M. Gobec S. Blanot D. (2008). Cytoplasmic steps of peptidoglycan biosynthesis. FEMS Microbiol. Rev. 32, 168–207. doi: 10.1111/j.1574-6976.2008.00104.x 18266853

[B11] Barros M. B. D. L. de Almeida Paes R. Schubach A. O. (2011). *Sporothrix schenckii* and sporotrichosis. Clin. Microbiol. Rev. 24, 633–654. doi: 10.1128/CMR.00007-11 21976602 PMC3194828

[B12] Bartnicki-Garcia S. Persson J. Chanzy H. (1994). An electron microscope and electron diffraction study of the effect of calcofluor and Congo red on the biosynthesis of chitin *in vitro* . Arch. Biochem. Biophys. 310, 6–15. doi: 10.1006/abbi.1994.1133 8161221

[B13] Bearne S. L. (1996). Active site-directed inactivation of *Escherichia coli* glucosamine-6-phosphate synthase: determination of the fructose 6-phosphate binding constant using a carbohydrate-based inactivator. J. Biol. Chem. 271, 3052–3057. doi: 10.1016/S0021-9258(18)97977-9 8621700

[B14] Beauvais A. Latgé J. P. (2018). Fungal cell wall. J. Fungi 4, 91. doi: 10.3390/jof4030091 PMC6162784 30081570

[B15] Bermejo C. Garcia R. Straede A. Rodriguez-Pena J. M. Nombela C. Heinisch J. J.. (2010). Characterization of sensor-specific stress response by transcriptional profiling of wsc1 and mid2 deletion strains and chimeric sensors in *Saccharomyces cerevisiae* . Omics J. Integr. Biol. 14, 679–688. doi: 10.1089/omi.2010.0060 20958245

[B16] Borgia P. T. (1992). Roles of the orlA, tsE, and bimG genes of *Aspergillus nidulans* in chitin synthesis. J. Bacteriol. 174, 384–389. doi: 10.1128/jb.174.2.384-389.1992 1309526 PMC205728

[B17] Broschat K. O. Gorka C. Page J. D. Martin-Berger C. L. Davies M. S. Huang H. C.. (2002). Kinetic characterization of human glutamine-fructose-6-phosphate amidotransferase I: potent feedback inhibition by glucosamine 6-phosphate. J. Biol. Chem. 277, 14764–14770. doi: 10.1074/jbc.M201056200 11842094

[B18] Calonge T. M. Nakano K. Arellano M. Arai R. Katayama S. Toda T.. (2000). *Schizosaccharomyces pombe* Rho2p GTPase regulates cell wall α-glucan biosynthesis through the protein kinase Pck2p. Mol. Biol. Cell 11, 4393–4401. doi: 10.1091/mbc.11.12.4393 11102532 PMC15081

[B19] Carvalho V. S. Gómez-Delgado L. Curto M.Á. Moreno M. B. Pérez P. Ribas J. C.. (2021). Analysis and application of a suite of recombinant endo-β (1, 3)-d-glucanases for studying fungal cell walls. Microb. Cell Fact. 20, 1–16. doi: 10.1186/s12934-021-01616-0 34217291 PMC8254974

[B20] Chakrabarti A. Bonifaz A. Gutierrez-Galhardo M. C. Mochizuki T. Li S. (2015). Global epidemiology of sporotrichosis. Med. Mycol. 53, 3–14. doi: 10.1093/mmy/myu062 25526781

[B21] Chattaway F. W. Holmes M. R. Barlow A. J. E. (1968). Cell wall composition of the mycelial and blastospore forms of *Candida albicans* . Microbiology 51, 367–376. doi: 10.1099/00221287-51-3-367 5657261

[B22] Chiew Y. Y. Shepherd M. G. Sullivan P. A. (1980). Regulation of chitin synthesis during germ-tube formation in *Candida albicans* . Arch. Microbiol. 125, 97–104. doi: 10.1007/BF00403204 6446267

[B23] Chmara H. Andruszkiewicz R. Borowski E. (1986). Inactivation of glucosamine-6-phosphate synthetase from *Salmonella typhimurium* LT2 by fumaroyl diaminopropanoic acid derivatives, a novel group of glutamine analogs. Biochim. Biophys.Acta Protein Struct. Mol. Enzym. 870, 357–366. doi: 10.1016/0167-4838(86)90240-2 3082365

[B24] Cifuentes B. Vicente C. (1982). Purification and properties of glucosaminephosphate isomerase of *Proteus mirabilis* . Z. Naturforsch. C 37, 381–384. doi: 10.1515/znc-1982-5-606 7051594

[B25] Conceição-Silva F. Morgado F. N. (2018). Immunopathogenesis of human sporotrichosis: what we already know. J. Fungi 4, 89. doi: 10.3390/jof4030089 PMC6162489 30065160

[B26] Coronado J. E. Mneimneh S. Epstein S. L. Qiu W. G. Lipke P. N. (2007). Conserved processes and lineage-specific proteins in fungal cell wall evolution. Eukaryot. Cell 6, 2269–2277. doi: 10.1128/EC.00044- 17951517 PMC2168262

[B27] Cortes J. C. G. Curto M.Á. Carvalho V. S. Perez P. Ribas J. C. (2019). The fungal cell wall as a target for the development of new antifungal therapies. Biotechnol. Adv. 37, 107352. doi: 10.1016/j.biotechadv.2019.02.008 30797093

[B28] Durand P. Golinelli-Pimpaneau B. Mouilleron S. Badet B. Badet-Denisot M. A. (2008). Highlights of glucosamine-6P synthase catalysis. Arch. Biochem. Biophys. 474, 302–317. doi: 10.1016/j.abb.2008.01.026 18279655

[B29] Durden F. M. Elewski B. (1997). Fungal infections in HIV-infected patients. Semin. Cutan. Med. Surg. 3, 200–212. doi: 10.1016/s1085-5629(97)80043-0 9300631

[B30] Dutka-Malen S. Mazodier P. Badet B. (1988). Molecular cloning and overexpression of the glucosamine synthetase gene from *Escherichia coli* . Biochimie 70, 287–290. doi: 10.1016/0300-9084(88)90073-9 3134953

[B31] Elhasi T. Blomberg A. (2019). Integrins in disguise-mechanosensors in *Saccharomyces cerevisiae* as functional integrin analogues. Microbial Cell 6, 335–355. doi: 10.15698/mic2019.08.686 31404395 PMC6685044

[B32] Endo A. Kakiki K. Misato T. (1970). Feedback inhibition of l-glutamine d-fructose 6-phosphate amidotransferase by uridine diphosphate n-acetylglucosamine in *Neurospora crassa* . J. Bacteriol 103, 588–594. doi: 10.1128/jb.103.3.588-594.1970 5474877 PMC248130

[B33] Etchebehere L. C. Maia J. C. (1989). Phosphorylation-dependent regulation of amidotransferase during the development of *Blastocladiella emersonii* . Arch. Biochem. Biophys. 272, 301–310. doi: 10.1016/0003-9861(89)90223-3 2546495

[B34] Fein M. Unkeless J. Chuang F. Sassaroli M. da Costa R. Väänänen H.. (1993). Lateral mobility of lipid analogues and GPI-anchored proteins in supported bilayers determined by fluorescent bead tracking. J. Membr. Biol. 135, 83–92. doi: 10.1007/BF00234654 8411132

[B35] Fèvre M. Girard V. Nodet P. (1990). “Cellulose and β-glucan synthesis in saprolegnia,” in Biochemistry of cell walls and membranes in fungi, vol. 97-107. (Berlin, Heidelberg: Springer). doi: 10.1007/978-3-642-74215-6_7

[B36] Free S. J. (2013). Fungal cell wall organization and biosynthesis. Adv. Genet. 81, 33–82. doi: 10.1016/B978-0-12-407677-8.00002-6 23419716

[B37] Frisa P. S. Sonneborn D. R. (1982). Developmentally regulated interconversions between end product-inhibitable and noninhibitable forms of a first pathway-specific enzyme activity can be mimicked *in vitro* by protein dephosphorylation-phosphorylation reactions. Proc. Nat. Acad. Sci. U.S.A. 79, 6289–6293. doi: 10.1073/pnas.79.20.6289 PMC347106 6959119

[B38] Garciía R. Bermejo C. Grau C. Peírez R. Rodriíguez-Penãa J. M. Francois J.. (2004). The global transcriptional response to transient cell wall damage in *Saccharomyces cerevisiae* and its regulation by the cell integrity signaling pathway. J. Biol. Chem. 279, 15183–15195. doi: 10.1074/jbc.M312954200 14739279

[B39] Ghosh S. Blumenthal H. J. Davidson E. Roseman S. (1960). Glucosamine metabolism: V. enzymatic synthesis of glucosamine 6-phosphate. J. Biol. Chem. 235, 1265–1273. doi: 10.1016/S0021-9258(18)69397-4 13827775

[B40] González-Ibarra J. Milewski S. Villagómez-Castro J. C. Cano-Canchola C. López-Romero E. (2010). *Sporothrix schenckii*: purification and partial biochemical characterization of glucosamine-6-phosphate synthase, a potential antifungal target. Med. Mycol. 48, 110–121. doi: 10.3109/13693780902856030 19353425

[B41] Gow N. A. Latge J. P. Munro C. A. (2017). The fungal cell wall: structure, biosynthesis, and function. Microbiol. Spectr. 5, 1–25. doi: 10.1128/microbiolspec.FUNK-0035-2016 PMC11687499 28513415

[B42] Gualtieri T. Ragni E. Mizzi L. Fascio U. Popolo L. (2004). The cell wall sensor Wsc1p is involved in reorganization of actin cytoskeleton in response to hypo-osmotic shock in *Saccharomyces cerevisiae* . Yeast 21, 1107–1120. doi: 10.1002/yea.1155 15484288

[B43] Heinisch J. J. Lorberg A. Schmitz H. P. Jacoby J. J. (1999). The protein kinase c-mediated MAP kinase pathway involved in the maintenance of cellular integrity in *Saccharomyces cerevisiae* . Mol. Microbiol. 32, 671–680. doi: 10.1046/j.1365-2958.1999.01375.x 10361272

[B44] Heller H. M. Fuhrer J. (1991). Disseminated sporotrichosis in patients with AIDS: case report and review of the literature. AIDS (London England) 5, 1243–1246. doi: 10.1097/00002030-199110000-00014 1664732

[B45] He D. Zhang X. Gao S. You H. Zhao Y. Wang L. (2021). Transcriptome analysis of dimorphic fungus *Sporothrix schenckii* exposed to temperature stress. Int. Microbiol. 24, 25–35. doi: 10.1007/s10123-020-00136-y 32691258 PMC7873001

[B46] Hosoi K. Kobayashi S. Ueha T. (1978). Sex difference in l-glutamine d-fructose-6-phosphate aminotransferase activity of mouse submandibular gland. Biochim. Biophys. Acta 543, 283–292. doi: 10.1016/0304-4165(78)90046-6 708787

[B47] Huang L. Zhang J. Song T. Yuan L. Zhou J. Yin H.. (2016). Antifungal curcumin promotes chitin accumulation associated with decreased virulence of *Sporothrix schenckii* . Int. Immunopharmacol. 34, 263–270. doi: 10.1016/j.intimp.2016.03.010 26995026

[B48] Ibe C. Munro C. A. (2021). Fungal cell wall: An underexploited target for antifungal therapies. PloS Pathog. 17, e1009470. doi: 10.1371/journal.ppat.1009470 33886695 PMC8061829

[B49] Ichinomiya M. Uchida H. Koshi Y. Ohta A. Horiuchi H. (2007). A protein kinase c-encoding gene, pkcA, is essential to the viability of the filamentous fungus *Aspergillus nidulans* . Biosci. Biotechnol. Biochem. 71, 2787–2799. doi: 10.1271/bbb.70409 17986778

[B50] Janiak A. M. Hoffmann M. Milewska M. J. Milewski S. (2003). Hydrophobic derivatives of 2-amino-2-deoxy-D-glucitol-6-phosphate: a new type of d-glucosamine-6-phosphate synthase inhibitors with antifungal action. Bioorg. Med. Chem. 11, 1653–1662. doi: 10.1016/S0968-0896(03)00049-X 12659751

[B51] Jiménez-Gutiérrez E. Alegría-Carrasco E. Alonso-Rodríguez E. Fernández-Acero T. Molina M. Martín H. (2020b). Rewiring the yeast cell wall integrity (CWI) pathway through a synthetic positive feedback circuit unveils a novel role for the MAPKKK Ssk2 in CWI pathway activation. FEBS J. 287, 4881–4901. doi: 10.1111/febs.15288 32150787

[B52] Jiménez-Gutiérrez E. Alegría-Carrasco E. Sellers-Moya Á. Molina M. Martín H. (2020a). Not just the wall: the other ways to turn the yeast CWI pathway on. Int. Microbiol. 23, 107–119. doi: 10.1007/s10123-019-00092-2 31342212

[B53] Jung U. S. Levin D. E. (1999). Genome-wide analysis of gene expression regulated by the yeast cell wall integrity signalling pathway. Mol. Microbiol. 34, 1049–1057. doi: 10.1046/j.1365-2958.1999.01667.x 10594829

[B54] Kapteyn J. C. Ram A. F. Groos E. M. Kollar R. Montijn R. C. Van Den Ende H.. (1997). Altered extent of cross-linking of beta1, 6-glucosylated mannoproteins to chitin in *Saccharomyces cerevisiae* mutants with reduced cell wall beta1, 3-glucan content. J. Bacteriol. 179, 6279–6284. doi: 10.1128/jb.179.20.6279-6284.1997 9335273 PMC179540

[B55] Katayama S. Hirata D. Arellano M. Pérez P. Toda T. (1999). Fission yeast α-glucan synthase Mok1 requires the actin cytoskeleton to localize the sites of growth and plays an essential role in cell morphogenesis downstream of protein kinase c function. J. Cell Biol. 144, 1173–1186. doi: 10.1083/jcb.144.6.1173 10087262 PMC2150588

[B56] Kenig M. Vandamme E. Abraham E. P. (1976). The mode of action of bacilysin and anticapsin and biochemical properties of bacilysin-resistant mutants. Microbiology 94, 46–54. doi: 10.1099/00221287-94-1-46 819624

[B57] Kikuchi H. Tsuiki S. (1976). Glucosaminephosphate synthase of human liver. Biochim. Biophys. Acta Enzymol. 422, 241–246. doi: 10.1016/0005-2744(76)90023-1 1247594

[B58] Klis F. M. Mol P. Hellingwerf K. Brul S. (2002). Dynamics of cell wall structure in *Saccharomyces cerevisiae* . FEMS Microbiol. Rev. 26, 239–256. doi: 10.1111/j.1574-6976.2002.tb00613.x 12165426

[B59] Kollár R. Reinhold B. B. Petráková E. Yeh H. J. Ashwell G. Drgonová J.. (1997). Architecture of the yeast cell wall: β (1→ 6)-glucan interconnects mannoprotein, β (1→ 3)-glucan, and chitin. J. Biol. Chem. 272, 17762–17775. doi: 10.1074/jbc.272.28.17762 9211929

[B60] Kornfeld R. (1967). Studies on l-glutamine d-fructose 6-phosphate amidotransferase: I. feedback inhibition by uridine diphosphate-n-acetylglucosamine. J. Biol. Chem. 242, 3135–3141. doi: 10.1016/S0021-9258(18)95943-0 4961641

[B61] Krynski S. Borowski E. Kuchta A. Borowski J. Becla E. (1952). Tetaine, a new antibiotic from *Bacillus pumilus* strains. Bull. State Inst. Mar. Trop. Med. 4, 310–318. 13009425

[B62] Kumamoto C. A. (2008). Molecular mechanisms of mechanosensing and their roles in fungal contact sensing. Nat. Rev. Microbiol. 6, 667–673. doi: 10.1038/nrmicro1960 18679170 PMC2760928

[B63] Lagorce A. Hauser N. C. Labourdette D. Rodriguez C. Martin-Yken H. Arroyo J.. (2003). Genome-wide analysis of the response to cell wall mutations in the yeast *Saccharomyces cerevisiae* . J. Biol. Chemi. 278, 20345–20357. doi: 10.1074/jbc.M211604200 12644457

[B64] Lagorce A. Le Berre-Anton V. Aguilar-Uscanga B. Martin-Yken H. Dagkessamanskaia A. Francois J. (2002). Involvementof GFA1, which encodes glutamine-fructose-6-phosphate amidotransferase, in the activation of chitin synthesis pathway in. Eur. J. Biochem. 269, 1607–1707. doi: 10.1046/j.1432-1327.2002.02814.x 11895440

[B65] Latgé J. P. (2010). Tasting the fungal cell wall. Cell. Microbiol. 12, 863–872. doi: 10.1111/j.1462-5822.2010.01474.x 20482553

[B66] Lloyd K. O. Bitoon M. A. (1971). Isolation and purification of a peptido-rhamnomannan from the yeast form of *Sporothrix schenckii.* structural and immunochemical studies. J. Immunol. 107, 663–671. 4999090

[B67] Lopes-Bezerra L. M. (2011). *Sporothrix schenckii* cell wall peptidorhamnomannans. Front. Microbiol. 2, 1–4. doi: 10.3389/fmicb.2011.00243 22203817 PMC3243909

[B68] Lopes-Bezerra L. M. Mora-Montes H. M. Zhang Y. Nino-Vega G. Rodrigues A. M. De Camargo Z. P.. (2018a). Sporotrichosis between 1898 and 2017: The evolution of knowledge on a changeable disease and on emerging etiological agents. Med. Mycol. 56, S126–S143. doi: 10.1093/mmy/myx103 29538731

[B69] Lopes-Bezerra L. M. Walker L. A. Nino-Vega G. Mora-Montes H. M. Neves G. W. Villalobos-Duno. (2018b). Cell walls of the dimorphic fungal pathogens *Sporothrix schenckii* and *Sporothrix brasiliensis* exhibit bilaminate structures and sloughing of extensive and intact layers. PloS Negl. Trop. Dis. 12, e0006169. doi: 10.1371/journal.pntd.0006169 29522522 PMC5903669

[B70] López-Romero E. Reyes-Montes M. D. R. Pérez-Torres A. Ruiz-Baca E. Villagómez-Castro J. C. Mora-Montes H. M.. (2011). *Sporothrix schenckii* complex and sporotrichosis, an emerging health problem. Future Microbiol. 6, 85–102. doi: 10.2217/fmb.10.157 21162638

[B71] Lucero H. A. Robbins P. W. (2004). Lipid rafts–protein association and the regulation of protein activity. Arch. Biochem. Biophys. 426, 208–224. doi: 10.1016/j.abb.2004.03.020 15158671

[B72] Maia J. C. D. C. (1994). Hexosamine and cell wall biogenesis in the aquatic fungus *Blastocladiella emersonii* . FASEB J. 8, 848–853. doi: 10.1096/fasebj.8.11.8070634 8070634

[B73] McKnight G. L. Mudri S. L. Mathewes S. L. Traxinger R. R. Marshall S. Sheppard P. O.. (1992). Molecular cloning, cDNA sequence, and bacterial expression of human glutamine: fructose-6-phosphate amidotransferase. J. Biol. Chem. 267, 25208–25212. doi: 10.1016/S0021-9258(19)74026-5 1460020

[B74] Michal G. Schomburg D. (2013). Biochemical pathways: An atlas of biochemistry and molecular biology. Wiley. p. 48–55

[B75] Milewski S. (2002). Glucosamine-6-phosphate synthase–the multi-facets enzyme. Biochim. Biophys. Acta Protein Struct. Mol. Enzymol. 1597, 173–192. doi: 10.1016/S0167-4838(02)00318-7 12044898

[B76] Milewski S. Chmara H. Andruszkiewicz R. Borowski E. Zaremba M. Borowski J. (1988). Antifungal peptides with novel specific inhibitors of glucosamine 6-phosphate synthase. Drugs Exp. Clin. Res. 14, 461–465. 3149235

[B77] Milewski S. Gabriel I. Olchowy J. (2006). Enzymes of UDP-GlcNAc biosynthesis in yeast. Yeast 23, 1–14. doi: 10.1002/yea.1337 16408321

[B78] Milewski S. Kuszczak D. Jedrzejczak R. Smith R. J. Brown A. J. Gooday G. W. (1999). Oligomeric structure and regulation of *Candida albicans* glucosamine-6-phosphate synthase. J. Biol. Chem. 274, 4000–4008. doi: 10.1074/jbc.274.7.4000 9933591

[B79] Milewski S. Smith R. J. Brown A. J. P. Gooday G. W. (1996). Purification and characterization of glucosamine-6-P synthase from *Saccharomyces cerevisiae* . Adv. Chitin Sci. 96–101.

[B80] Miyagi T. Tsuiki S. (1974). Comparison of the properties of glucosaminephosphate isomerase (glutamine-forming) from rat liver and a hepatoma. Biochim. Biophys. Acta Enzymol. 358, 144–153. doi: 10.1016/0005-2744(74)90266-6 4368527

[B81] Molina M. Gil C. Pla J. Arroyo J. Nombela C. (2000). Protein localisation approaches for understanding yeast cell wall biogenesis. Microsc. Res. Tech. 51, 601–612. doi: 10.1002/1097-0029(20001215)51:6<601::AID-JEMT9>3.0.CO;2-I 11169861

[B82] Neuss N. Molloy B. B. Shah R. DeLaHiguera N. (1970). The structure of anticapsin, a new biologically active metabolite of *Streptomyces griseoplanus* . Biochem. J. 118, 571–575. doi: 10.1042/bj1180571 5481496 PMC1179253

[B83] Nodet P. Capellano A. Fvre M. (1990a). Morphogenetic effects of Congo red on hyphal growth and cell wall development of the fungus *Saprolegnia monoica* . J. Gen. Microbiol. 136, 303–310. doi: 10.1099/00221287-136-2-303

[B84] Nodet P. Girard V. Fvre M. (1990b). Congo Red inhibits *in vitro* β-glucan synthases of *Saprolegnia* . FEMS Microbiol. Lett. 69, 225–228. doi: 10.1111/j.1574-6968.1990.tb04234.x

[B85] Nohe A. Keating E. Fivaz M. van der Goot F. G. Petersen N.O (2006). Dynamics of GPI-anchored proteins on the surface of living cells. Nanotechnol. Biol. Med. 2, 1–7. doi: 10.1016/j.nano.2005.10.013 17292110

[B86] Norrman J. Giddings T. H. Cantino E. C. (1975). Partial purification and properties of l-glutamine: d-fructose 6-phosphate aminotransferase from zoospores of blastocladiella emersonii. Phytochemistry 14, 1271–1274. doi: 10.1016/S0031-9422(00)98608-4 4692652

[B87] Nowak-Jary J. Andruszkiewicz R. (2009). Antifungal activity of thionated analogues of nva-FMDP and lys-Nva-FMDP. Pol. J. Microbiol. 58, 295–299. 20380139

[B88] Orlean P. (1997). Biogenesis of yeast wall surface components. Biology: Cold Spring Harbor Monograph Arch. 21, 229–362. doi: 10.1101/087969364.21C.229

[B89] Ortiz-Ramírez J. A. Cuéllar-Cruz M. López-Romero E. (2021). Responses of *Sporothrix globosa* to the cell wall perturbing agents Congo red and calcofluor white. Antonie van Leeuwenhoek 114, 609–624. doi: 10.1007/s10482-021-01545-3 33660079

[B90] Popolo L. Gualtieri T. Ragni E. (2001). The yeast cell-wall salvage pathway. Med. Mycol. 39, 111–121. doi: 10.1080/mmy.39.1.111.121 11800265

[B91] Previato J. O. Gorin P. A. Travassos L. R. (1979). Cell wall composition in different cell types of the dimorphic species *Sporothrix schenckii* . Exp. Mycol. 3, 83–91. doi: 10.1016/S0147-5975(79)80020-1

[B92] Queiroz-Telles F. Buccheri R. Benard G. (2019). Sporotrichosis in immunocompromised hosts. J. Fungi 5, 8. doi: 10.3390/jof5010008 PMC6463096 30641918

[B93] Raczynska J. Olchowy J. Konariev P. V. Svergun D. I. Milewski S. Rypniewski W. (2007). The crystal and solution studies of glucosamine-6-phosphate synthase from *Candida albicans* . J. Mol. Biol. 372, 672–688. doi: 10.1016/j.jmb.2007.07.002 17681543

[B94] Ram A. F. Arentshorst M. Damveld R. A. Klis F. M. van den Hondel C. A. (2004). The cell wall stress response in *Aspergillus niger* involves increased expression of the glutamine: fructose-6-phosphate amidotransferase-encoding gene (gfaA) and increased deposition of chitin in the cell wall. Microbiology 150, 3315–3326. doi: 10.1099/mic.0.27249-0 15470111

[B95] Ram A. F. Klis F. M. (2006). Identification of fungal cell wall mutants using susceptibility assays based on calcofluor white and Congo red. Nat. Protoc. 1, 2253–2256. doi: 10.1038/nprot.2006.397 17406464

[B96] Rapp C. Jung G. Katzer W. Loeffler W. (1988). Chlorotetain from *Bacillus subtilis*, an antifungal dipeptide with an unusual chlorine-containing amino acid. Angew. Chem. Int. Ed. Engl. 27, 1733–1734. doi: 10.1002/anie.198817331

[B97] Rogers H. J. Newton G. G. F. Abraham E. P. (1965). Production and purification of bacilysin. Biochem. J. 97, 573–578. doi: 10.1042/bj0970573 16749167 PMC1264678

[B98] Romeo O. Criseo G. (2013). What lies beyond genetic diversity in *Sporothrix schenckii* species complex? new insights into virulence profiles, immunogenicity and protein secretion in *S. schenckii sensu stricto* isolates. Virulence 4, 203–206. doi: 10.4161/viru.23467 23334066 PMC3711977

[B99] Roncero C. Duran A. (1985). Effect of calcofluor white and Congo red on fungal cell wall morphogenesis: *in vivo* activation of chitin polymerization. J. Bacteriol. 163, 1180–1185. doi: 10.1128/jb.163.3.1180-1185.1985 3897187 PMC219256

[B100] Sachadyn P. Jędrzejczak R. Milewski S. Kur J. Borowski E. (2000). Purification to homogeneity of *Candida albicans* glucosamine-6-phosphate synthase overexpressed in *Escherichia coli* . Protein Expr. Purif. 19, 343–349. doi: 10.1006/prep.2000.1253 10910723

[B101] Sánchez-López J. F. González-Ibarra J. Álvarez-Vargas A. Milewski S. Villagómez-Castro J. C. Cano-Canchola C.. (2015). Isolation of the GFA1 gene encoding glucosamine-6-phosphate synthase of *Sporothrix schenckii* and its expression in *Saccharomyces cerevisiae* . Protein Expr. Purif 110, 57–64. doi: 10.1016/j.pep.2014.12.002 25514203

[B102] Sánchez-López J. F. González-Ibarra J. Macías-Segoviano J. I. Cuéllar-Cruz M. Álvarez-Vargas A. Cano-Canchola C.. (2019). Congo Red affects the growth, morphology and activity of glucosamine-6-phosphate synthase in the human pathogenic fungus *Sporothrix schenckii* . Arch. Microbiol. 201, 135–141. doi: 10.1007/s00203-018-1576-1 30302500

[B103] Sanz A. B. García R. Rodríguez-Peña J. M. Arroyo J. (2017). The CWI pathway: regulation of the transcriptional adaptive response to cell wall stress in yeast. J. Fungi 4, 1–12. doi: 10.3390/jof4010001 PMC5872304 29371494

[B104] Schuster M. Martin-Urdiroz M. Higuchi Y. Hacker C. Kilaru S. Gurr S. J.. (2016). Co-Delivery of cell-wall-forming enzymes in the same vesicle for coordinated fungal cell wall formation. Nat. Microbiol. 1, 1–11. doi: 10.1038/nmicrobiol.2016.149 27563844

[B105] Selitrennikoff C. P. (1984). Calcofluor white inhibits *Neurospora* chitin synthetase activity. Exp. Mycol. 8, 269–272. doi: 10.1016/0147-5975(84)90011-2

[B106] Selitrennikoff C. P. Dalley N. E. Sonneborn D. R. (1980). Regulation of the hexosamine biosynthetic pathway in the water mold *Blastocladiella emersonii*: sensitivity to endproduct inhibition is dependent upon the life cycle phase. Proc. Nat. Acad. Sci. U.S.A. 77, 5998–6002. doi: 10.1073/pnas.77.10.5998 PMC350200 16592895

[B107] Shepherd M. G. Ghazali H. M. Sullivan P. A. (1980). N-acetyl-d-glucosamine kinase and germ-tube formation in *Candida albicans* . Exp. Mycol. 4, 147–159. doi: 10.1016/0147-5975(80)90018-3

[B108] Smith C. A. (2006). Structure, function, and dynamics in the mur family of bacterial cell wall ligases. J. Mol. Biol. 362, 640–655. doi: 10.1016/J.JMB.2006.07.066 16934839

[B109] Smith R. J. Milewski S. Brown A. J. Gooday G. W. (1996). Isolation and characterization of the GFA1 gene encoding the glutamine: fructose-6-phosphate amidotransferase of *Candida albicans* . J. Bacteriol. 178, 2320–2327. doi: 10.1128/jb.178.8.2320-2327.1996 8636033 PMC177940

[B110] Smits G. J. Kapteyn J. C. van den Ende H. Klis F. M. (1999). Cell wall dynamics in yeast. Curr. Opin. Microbiol. 2, 348–352. doi: 10.1016/S1369-5274(99)80061-7 10458981

[B111] Teixeira M. D. M. Rodrigues A. M. Tsui C. K. de Almeida L. G. P. Van Diepeningen A. D. van den Ende B. G.. (2015). Asexual propagation of a virulent clone complex in a human and feline outbreak of sporotrichosis. Eukaryot. Cell 14, 158–169. doi: 10.1128/EC.00153-14 25480940 PMC4311920

[B112] Teplyakov A. Leriche C. Obmolova G. Badet B. Badet-Denisot M. A. (2002). From lobry de bruyn to enzyme-catalyzed ammonia channelling: molecular studies of d-glucosamine-6P synthase. Nat. Prod. Rep. 19, 60–69. doi: 10.1039/B103713G 11902440

[B113] Teplyakov A. Obmolova G. Badet B. Badet-Denisot M. A. (2001). Channeling of ammonia in glucosamine-6-phosphate synthase. J. Mol. Biol. 313, 1093–1102. doi: 10.1006/jmbi.2001.5094 11700065

[B114] Travassos L. R. (1985). “Sporothrix schenckii,” in Fungal dimorphism (Boston, MA: Springer), 121–163. doi: 10.1007/978-1-4684-4982-2_6

[B115] Travassos L. R. Lloyd K. O. (1980). *Sporothrix schenckii* and related species of *Ceratocystis* . Microbiol. Rev. 44, 683–721. doi: 10.1128/mr.44.4.683-721.1980 7010114 PMC373199

[B116] Valdivia R. H. Schekman R. (2003). The yeasts Rho1p and Pkc1p regulate the transport of chitin synthase III (Chs3p) from internal stores to the plasma membrane. Proc. Nat. Acad. Sci. U.S.A. 100, 10287–10292. doi: 10.1073/pnas.1834246100 PMC193553 12928491

[B117] Valle N. R. D. Rosario M. Torres-Blasini G. (1983). Effects of pH, temperature, aeration and carbon source on the development of the mycelial or yeast forms of *Sporothrix schenckii* from conidia. Mycopathologia 82, 83–88. doi: 10.1007/BF00437335 6888501

[B118] Vannini G. L. Poli F. Donini A. Pancaldi S. (1983). Effects of Congo red on wall synthesis and morphogenesis in *Saccharomyces cerevisiae* . Plant Sci. Lett. 31, 9–17. doi: 10.1016/0304-4211(83)90125-6

[B119] Vermeulen C. A. Wessels J. G. (1986). Chitin biosynthesis by a fungal membrane preparation: Evidence for a transient non-crystalline state of chitin. Eur. J. Biochem. 158, 411–415. doi: 10.1111/j.1432-1033.1986.tb09768.x 3732275

[B120] Vessal M. Hassid W. Z. (1972). Partial purification and properties of l-glutamine d-fructose 6-phosphate amidotransferase from *Phaseolus aureus* . Plant Physiol. 49, 977–981. doi: 10.1104/pp.49.6.977 16658095 PMC366091

[B121] Villalobos-Duno H. L. Barreto L. A. Alvarez-Aular Á. Mora-Montes H. M. Lozoya-Pérez N. E. Franco B.. (2021). Comparison of cell wall polysaccharide composition and structure between strains of *Sporothrix schenckii* and *Sporothrix brasiliensis* . Front. Microbiol. 12. doi: 10.3389/fmicb.2021.726958 PMC8489378 34616384

[B122] Watzele G. Tanner W. (1989). Cloning of the glutamine: fructose-6-phosphate amidotransferase gene from yeast: pheromonal regulation of its transcription. J. Biol. Chem. 264, 8753–8758. doi: 10.1016/S0021-9258(18)81857-9 2656689

[B123] Wojciechowski M. Milewski S. Mazerski J. Borowski E. (2005). Glucosamine-6-phosphate synthase, a novel target for antifungal agents. molecular modelling studies in drug design. Acta Biochim. Pol. 52, 647–653. doi: 10.18388/abp.2005_3425 16082410

[B124] Yang N. Yu Z. Jia D. Xie Z. Zhang K. Xia Z.. (2014). The contribution of pir protein family to yeast cell surface display. Appl. Microbiol. Biotechnol. 98, 2897–2905. doi: 10.1007/s00253-014-5538-5 24493571

[B125] Zhang Y. Hagen F. Stielow B. Rodrigues A. M. Samerpitak K. Zhou X.. (2015). Phylogeography and evolutionary patterns in *Sporothrix* spanning more than 14 000 human and animal case reports. Pers. Mol. Phylogeny Evol. Fungi 35, 1–20. doi: 10.3767/003158515X687416 PMC4713101 26823625

[B126] Zwierz K. Gindzieński A. Popowicz J. (1978). Purification and properties of hexosamine isomerase (EC 3.5. 1.19) from pig intestinal mucosa. Enzyme 23, 339–345. doi: 10.1159/000458598 30620

